# Light-Controlled Anticancer Activity and Cellular Uptake of a Photoswitchable Cisplatin Analogue

**DOI:** 10.1021/acs.jmedchem.4c01575

**Published:** 2024-10-24

**Authors:** Marta Stolarek, Kamil Kaminski, Marta Kaczor-Kamińska, Magdalena Obłoza, Piotr Bonarek, Anna Czaja, Magdalena Datta, Wojciech Łach, Mateusz Brela, Artur Sikorski, Janusz Rak, Maria Nowakowska, Krzysztof Szczubiałka

**Affiliations:** † Faculty of Chemistry, Jagiellonian University, Gronostajowa 2, 30-387 Cracow, Poland; ‡ Jagiellonian University, Doctoral School of Exact and Natural Sciences, Łojasiewicza 11, 30-348 Cracow, Poland; § Chair of Medical Biochemistry, Jagiellonian University, Collegium Medicum, Kopernika 7C, 31-034 Cracow, Poland; ∥ Faculty of Biochemistry, Biophysics and Biotechnology, Jagiellonian University, Gronostajowa 7, 30-387 Cracow, Poland; ⊥ Faculty of Chemistry, University of Gdańsk, Wita Stwosza 63, 80-308 Gdansk, Poland

## Abstract

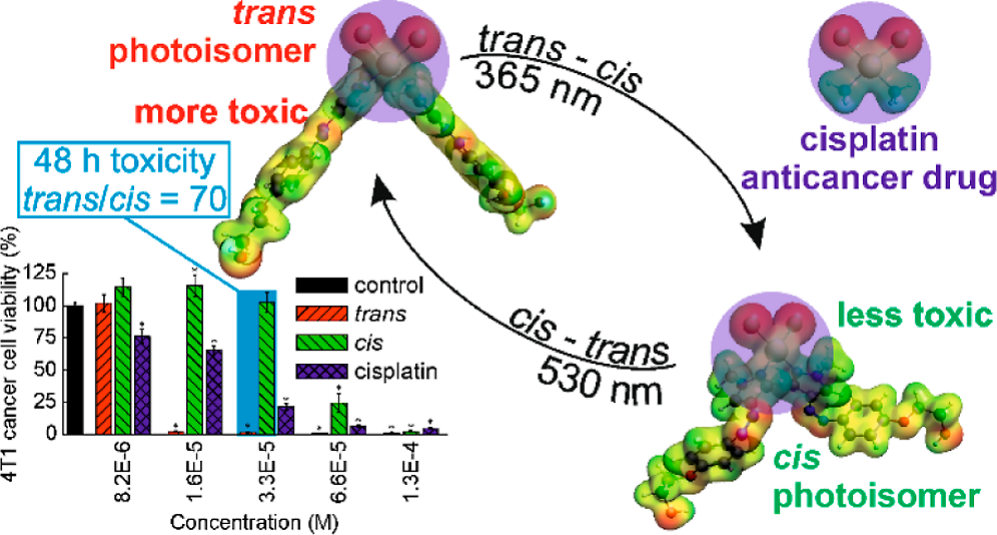

A photoactive analogue of cisplatin was synthesized with two arylazopyrazole ligands, able to undergo *trans*–*cis*/*cis*–*trans* photoisomerizations. The *cis* photoisomer showed a dark half-life of 9 days. The cytotoxicities of both photoisomers of the complex were determined in several cancer and normal cell lines and compared to that of cisplatin. The *trans* photoisomer of the complex was much more cytotoxic than both the *cis* photoisomer and cisplatin, and was more toxic for cancer (4T1) than for normal (NMuMG) murine breast cells. 4T1 cell death occurred through necrosis. Photoisomerization of the *trans* and *cis* photoisomers internalized by the 4T1 cells increased and decreased their viability, respectively. The cellular uptake of the *trans* photoisomer was stronger than that of both the *cis* photoisomer and cisplatin. Both photoisomers interacted with DNA faster than cisplatin. The *trans* photoisomer was bound stronger by bovine serum albumin and induced a greater decrease in cellular glutathione levels than the *cis* photoisomer.

## Introduction

Cisplatin (*cis*-diamminedichloroplatinum(II), CDDP), a planar complex composed of a Pt(II) cation coordinating two chloride ions and two ammonia molecules, was first described by Michele Peyrone almost 200 years ago.^1^ In 1965, Rosenberg et al. found its strong bacteriostatic properties,^2^ but soon they also realized its anticancer potential. Its application in Swiss white mice with sarcoma resulted in the tumor remission and survival of the animals.^3^ After 6 months of the treatment, the mice did not show any signs of cancer. In 1978, cisplatin was approved by the FDA and a year later in Europe. Tests of over 4000 platinum compounds resulted in the introduction of two other Pt(II) complexes, carboplatin and oxaliplatin, into the clinic. Now cisplatin and other platinum-based chemotherapeutics are mainstay first-line anticancer drugs used to treat almost 50% of all cancers.^4^

The mechanism of the anticancer activity of cisplatin is well recognized. Cisplatin enters cells via passive diffusion through the cell membrane^5^ or is actively transferred by the copper transporter protein (CTR1).^6^ Inside the cell, Cl^–^ ligands of cisplatin are replaced with water molecules forming a hydrated, positively charged complex. It reacts with two nucleobase moieties in the DNA, primarily in the same strand, forming a cyclic adduct^7^ which leads to cell apoptosis or necrosis.

Unfortunately, the therapeutic effects of cisplatin are hindered by its adverse effects, which can be both serious sequelae such as cardiotoxicity as well as milder symptoms such as nausea and vomiting.^8^ Also, the therapeutic efficiency of cisplatin is limited due to resistance to this drug, which results in the need to escalate the doses, aggravating adverse effects.

The possibility of improving the therapeutic effects and limiting the adverse effects of cisplatin is offered by the photopharmacological approach.^9^ It involves the application of a photoactive molecule, which itself is a drug (as opposed to the photosensitizers used in photodynamic therapy, PDT). It may be also attached to a drug molecule or may replace a structurally similar part of a drug molecule (the procedures known as “azo-extension” and “azologization”, respectively, in the case of azobenzene-based photoactive compounds^10,11^). The pharmacological activity of such photoactive systems can be modified by absorption of light followed by irreversible cleavage (termed as “uncaging”, “photoactivation”, or “photocleavage”) of a bond and release of an activated (or inactivated) species.^12,13^ Light may also trigger a reversible change in the shape of the molecule, a photoswitch (PS), and its physicochemical properties, which in turn may change (increase or decrease) its pharmacological activity.^10,11^ The opposite change of activity (decrease or increase, respectively) can be achieved by irradiation with light of another wavelength.

The discovery that the biological activity of the platinum compounds may be controlled with light has been made concurrently with the discovery of bacteriostatic activity of cisplatin by Rosenberg et al. who found that irradiation of a solution of PtCl_6_^2–^ and NH_4_^+^ ions with UV light leads to the formation of a mixture of different Pt(IV) complexes, of which *cis*-[PtCl_4_(NH_3_)_2_], i.e., cisplatin, shows bacteriostatic activity.^14^ Later, photoresponsible platinum compounds were synthesized based both as photocages and photoswitches.^15^ The first photoactive Pt(IV) compounds employing the former principle were the Pt(IV) iodides which upon irradiation for 6 h with >375 nm light released iodide ligands with the formation of a product that bound to DNA and was more cytotoxic to bladder cancer cells than the nonirradiated compound, bringing about a 22% enhancement in the antiproliferative activity.^16^ Pt(IV) photoactive complexes were extensively studied by Sadler et al. These compounds contained a diazido ligand which was released upon irradiation with a 420 nm light, thereby activating the molecule.^17−20^ Puddephatt et al. have thoroughly studied Pt(II) and Pt(IV) complexes, including those containing one photoisomerizable azobenzene-based moiety, however, not in the context of the photoswitchability of their biological properties.^21−24^ To the best of our knowledge, there are only a few papers on photoswitchable platinum complexes whose toxicity in cancer cells was studied. One of them describes a complex containing a photoswitchable 1,2-dithienylethene motif whose open and closed forms showed different cytotoxicity.^25^ Samper et al. obtained a Pt complex containing a photoswitchable azobenzene group; however, they did not find statistically significant differences between the cytotoxicities of both photoisomers.^26^

In this study, we have designed and synthesized a Pt complex that may be considered as an analogue of cisplatin in which both ammonia ligands were replaced with an arylazopyrazole (AAP)-based photoswitchable ligand (**1**) able to undergo *trans–cis* and *cis*–*trans* photoisomerizations, both with high yield. AAPs are a relatively new class of photoswitches offering several advantages over azobenzenes widely used so far.^27,28^ We have hypothesized that, on the one hand, the complex will be cytotoxic due to the presence of the cisplatin motif in its molecule (i.e., two hydrolyzable chloride ligands *cis*-coordinated to a Pt atom expected to give the complex the ability to cross-link DNA). On the other hand, the photoisomerization of two AAP ligands in the complex is accompanied by a remarkable change in the complex geometry, as shown by preliminary theoretical calculations. We expected that photoisomerization may result in a significant and reversible change in the complex toxicity which could be thus spatiotemporally controlled with light. We have positively verified these hypotheses in a series of *in vitro* experiments, including the determination of the cytotoxicity of the complex photoisomerized both before cellular uptake and upon internalization by the cells.

## Results and Discussion

### PS Design and Synthesis

A crucial step in the development of a photoswitchable cisplatin analogue was to design a PS ligand that could be easily coordinated by the Pt(II) ion to form a complex. It was assumed that the complex should significantly change its molecular shape and size and consequently physicochemical properties upon photoswitching, which would in turn result in the change of its biological properties. Finally, the photoswitch should have favorable photochemical properties, i.e., undergo fast, quantitative, and reversible photoswitching under irradiation with light of possibly long wavelength. Moreover, both photoisomers should be thermally stable in physiological conditions for a time long enough so that the difference in their biological properties, cytotoxicity in particular, could be detected, if any. Moreover, the PS should be nontoxic if released from the complex.

To fulfill as many as possible of these requirements, we considered arylazopyrazole (AAP)-based PSs as ligands. One of the two hetero nitrogen atoms in the pyrazole ring of AAPs can coordinate with the Pt(II) atom, forming a planar complex. The complexes formed between PtCl_4_^2–^ ions, used in our synthesis, and pyrazole and imidazole have preferentially *cis* geometry^29−31^ since the trans effect of pyrazole is weaker than that of the chloride anion.^32^ Thus, compounds containing pyrazole rings in their structure can form complexes which are analogues of cisplatin. On the other hand, it was recently found that pyrazolylazophenyl alkyl ethers, which are AAPs with phenyl ring substituted with alkoxy groups, have both photostationary states (PSS) composed of almost pure *trans* and *cis* photoisomers when irradiated with 365 and 530 nm light, respectively.^33^ Their thermally unstable *cis* photoisomers show exceptionally long half-lives (up to 3 months in organic media at room temperature).^33^ In fact, their *trans*–*cis* photoisomerization could be also achieved using a visible 400 nm light, although photoswitching was then not complete (PSS contained about 80% of the *cis* photoisomer^33^). Of potential importance were also a variety of beneficial pharmacological activities of pyrazole derivatives^34^ including antiviral,^35^ anti-inflammatory,^35^ antimicrobial,^36^ and, the most relevant from the point of view of this research, anticancer ones.^37^ Importantly, the N=N bond in AAPs, unlike that in the azobenzene-based PSs, shows excellent resistance to reduction with cellular glutathione (GSH).^27^

### Photoswitch Structure and Properties

**Figure 1:**
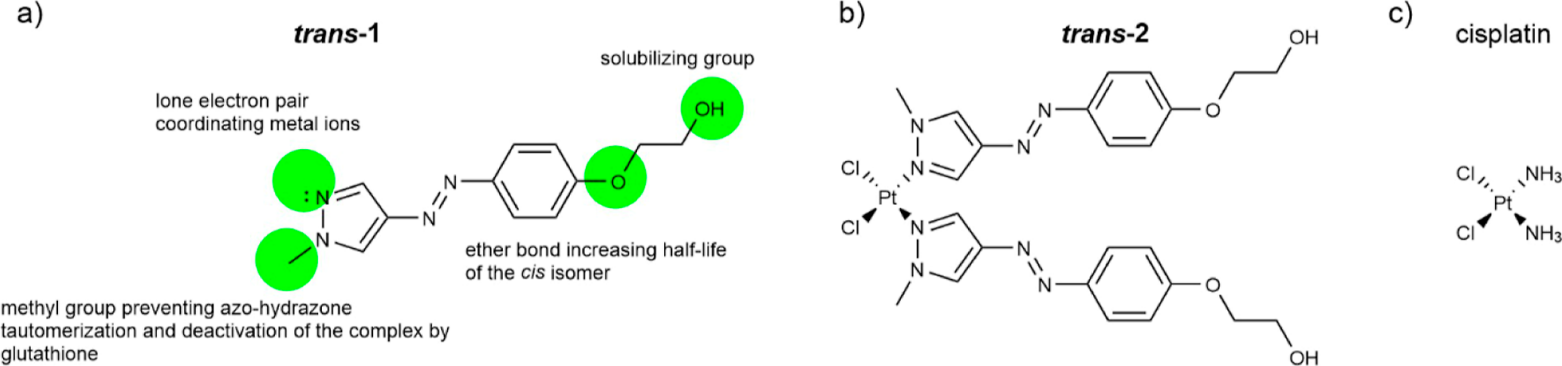
Structures of the (a) designed photoswitch (***trans*-1**) with important structural elements responsible for its favorable photopharmacological properties, (b) (***trans*-2**), and (c) cisplatin.

Considering the above facts, we have designed an AAP-based photoswitch (**1**, Figure 1a) with a –OCH_2_CH_2_OH substituent at the *para* position of the phenyl ring to achieve a long half-life of the *cis* photoisomer, characteristic of pyrazolylazophenyl alkyl ethers. The alkyl chain was terminated with a hydroxyl group and was possibly short to maximize the solubility of the complex in aqueous media. The pyrazolyl ring with a *N* atom substituted with a methyl group was used to additionally increase the half-life of the photoswitch (the presence of a N–H bond in unsubstituted pyrazole leads to fast *cis*–*trans* thermal isomerization through an azo-hydrazone tautomerization mechanism^38^). Moreover, the methyl group provided a steric hindrance close to the coordination center which had been found to protect the anticancer planar complexes of Pt(II) from the axial nucleophilic attack of the cellular thiols, GSH and metallothionein in particular, which could result in the complex inactivation and the development of drug resistance.^39^

The synthesis of ***trans*-1** was described in our previous work.^40^ Its structure was confirmed with EA, ^1^H NMR, ^13^C NMR,^40^ LC–MS,^40^ and UV–vis spectroscopy (see Table S1, Figure S3, and Figure S4 in the Supporting Information). ***trans*-1** was soluble in water well enough to give measurable UV–vis spectra (solubility in water 0.58 mg/mL, see Figure S20). The yield of the **1** photoisomerizations was estimated based on ^1^H NMR spectra. The spectrum of ***trans*-1** (nonirradiated) in DMF is shown in Figure S5a. The ***trans***-**1** solution was then irradiated with a 365 nm light to switch it to the *cis* configuration. The progress of the photoisomerization could also be followed using UV–vis spectra (Figure S4). The singlets of the pyrazole protons shifted significantly from δ = 8.40 and 7.91 ppm in ***trans*-1** to 7.89 and 6.48 ppm in ***cis*-1** (Figure S5b). The doublets of the phenylene protons shifted from 7.76, 7.74, 7.11, and 7.09 ppm in ***trans*-1** to 7.10, 7.08, 6.78, and 6.76 ppm in ***cis*-1**, respectively. Since the pyrazole and phenylene proton signals of ***trans***-**1** are absent in the spectrum of ***cis***-**1**, the *trans–cis* photoisomerization of ***trans*-1** under 365 nm light can be considered as quantitative (100%). Irradiation of ***cis*-1** with 530 nm light to reverse its configuration back to the *trans* one resulted in opposite shifts of the signals (Figure S5c); however, the presence of the residual signals of pyrazole and phenylene protons of ***cis***-**1** indicated that the *cis*–*trans* photoisomerization was not quantitative, but its yield, calculated based on the signal integration, was still very high (89%). Thus, the ^1^H NMR measurements confirmed the possibility to photoswitch **1** with high yield in both directions, which was a prerequisite for its application in the synthesis of the complex with photocontrollable biological activity.

### Photoswitchable Complex of Pt(II) with **1** and Cl^–^ as Ligands

**1** was used as a ligand to obtain a Pt(II) complex by substituting two Cl^–^ ligands in PtCl_4_^2–^ with two **1** molecules (Figure 1b). Dichloroplatinum Pt(II) complexes in either *cis* or *trans* configuration complexes can be obtained selectively, depending on the experimental conditions applied.^29−31,41^ The synthesis was based on the literature procedure,^41^ allowing formation of the dichloroplatinum Pt(II) complex with two highly substituted pyrazole derivative ligands in *cis* configuration which was proven by the authors with the Kurnakov test.^42^ The complex may be thus considered as an analogue of cisplatin (Figure 1c) with both ammonia molecules replaced with **1**. The complex is hereafter referred to as **2**, if the *cis*/*trans* configuration of **1** is irrelevant, while the complex, in which **1** is known to have *cis* or *trans* configuration, is denoted as ***cis*-2** and ***trans*-2**, respectively. The complex structure was confirmed/characterized by ^1^H NMR, ATR-FT-IR, and LC–MS techniques (see Figures S5d,e,f and S6–S11). The EA results confirmed the assumed elemental compositions of **1** and **2** (Tables S1 and S2), which are significantly different.

In the spectrum of the nonirradiated complex (i.e., ***trans*-2**) in DMF-*d*_7_, the most intense signals correspond to those of ***trans*-1** (compare Figure S5a,d). In the spectrum of the complex, both aromatic pyrazole singlets (at 8.81 and 8.79 ppm) and the methyl proton singlet (4.55 ppm) are significantly shifted downfield compared to their signals in ***trans*-1**. This is due to the proximity of the coordination center in the complex. On the other hand, the doublets of distant phenylene protons (7.76, 7.74, 7.12, and 7.10 ppm) and triplets of ethylene protons (4.15 and 3.85) are not shifted. The spectrum of the complex irradiated with a 365 nm light (i.e., ***cis*-2**) became more complicated (Figure S5e). Compared to the *trans* photoisomer of the complex, the pyrazole singlets in the *cis* photoisomer of the complex shifted upfield to 8.36 and 6.73 ppm, and the methyl singlet shifted upfield to 4.18 ppm. The position of these signals was shifted downfield compared to that of the respective signals of ***cis*-1** due to the proximity of the coordination center. The phenylene doublets appear at 7.03, 7.01, 6.79, and 6.76 and are accompanied by smaller signals (see below). Their position and that of the ethylene protons is similar to that in the free ***cis*-1** Based on the integration of the pyrazole proton signal of the *cis* and *trans* photoisomer (at 8.36 ppm and a residual signal at 8.79 ppm, respectively) in Figure S5f, the yield of the complex *trans–cis* photoisomerization could be estimated as at least 98%. After irradiation of the *cis* photoisomer of the complex with a 530 nm light, resulting in *cis*–*trans* photoisomerization, the positions of the main signals were the same as those of the nonirradiated complex (compare Figures S5f,d), except for the disappearance of the hydroxyl proton signal at 5.0 ppm. This confirms the possibility of achieving back-and-forth switching between *trans* and *cis* forms of the complex. The pyrazole proton signals of the *cis* photoisomer are completely absent in Figure S5f; thus *cis*–*trans* photoisomerization of the complex can be achieved with 100%, even higher than for **1** itself.

The presence of a singlet at 3.97 ppm in the spectrum of the nonirradiated complex (Figure S5d), characteristic of methyl protons of ***trans*-1** (Figure S5a), indicates that the complex contained about 3.8 mol % (about 1.2 wt %) of ***trans*-1** based on signal integration. The content of ***trans*-1** in the complex solution irradiated first with 365 nm and then with 530 nm light increased up to 9 mol % (about 3 wt %) (Figure S5f). This indicates that the complex underwent photodissociation to a small extent with the formation of free ***trans*-1** and the complex monosubstituted with ***trans*-1** and DMF molecules as ligands, i.e., *cis*-PtCl_2_(***trans*-1**) (DMF).^43^ The formation of *cis*-PtCl_2_(***trans*-1**) (DMF) may also explain the presence of a small singlet accompanying the pyrazole singlets (8.84 ppm) and two small doublets accompanying phenylene doublets (7.84 and 7.82; 7.16 and 7.13 ppm) of the *trans* complex photoisomer (Figures S5d,f). The small doublets (7.10 and 7.08; 6.87 and 6.84 ppm) are also present close to the phenylene doublets of the *cis* complex (Figure S5e). The fact that the position of the small doublets shifted after both irradiations and that their relative intensity increased after irradiation confirms the assumption that they originate from a monosubstituted complex. However, this increase is difficult to quantify due to the signal overlap and its low intensity.

The ATR-FT-IR spectrum of the complex is dominated by bands of the ***trans*-1** ligand (Figure S7). The ***trans*-1** bands do not shift due to complexation, or shifts are very small and occur in both directions. The relative intensities of some of the bands of the complex changed, as observed in analogous Pt(II) complexes with *N*-heteroaromatic compounds.^44^ The bands characteristic of Pt–Cl stretching vibrations occur in the far-infrared region (450–100 cm^–1^),^44^ outside of the accessible wavenumber range of the apparatus. The LC chromatogram (Figure S8a) reveals a single dominant signal at a retention time of 5.73 min. In the mass spectrum of the compound showing this LC signal (Figure S8b), the peaks with the highest *m*/*z* values are grouped around *m*/*z* of 757–762 which agrees with the assumed molecular weight of the complex of 758.82 Da. The identity of the complex is additionally supported by the presence of groups of signals around *m*/*z* of 722–725 and 686–689, corresponding to the complex with one and two Cl atoms detached, respectively (Figure S8b), and by a signal at *m*/*z* of 247.13 corresponding to the detached protonated **1** ligand (molecular weight 246.27 Da). The presence of weak signals close to the complex signal and a weak signal at 4.67 min in the chromatogram, whose mass spectrum features a single signal at *m*/*z* of 247.26 (Figure S8c) corresponding to **1**, may be due to the fact that the complexes of Pt(II) with pyrazoles may partially degrade on interaction with the stationary phase of the chromatographic columns, as observed by others.^30,41^ We also observed that the complex is susceptible to degradation in different analytical conditions (e.g., when analyzed using other columns, data not shown). The region of the HR-MS spectrum corresponding to the molecular ion of the complex showed an isotope pattern (Figure S9a) characteristic of a compound with one Pt and two Cl atoms (Figure S9b), confirming the predicted composition of the complex. In spite of intensive efforts, so far we have not succeeded in obtaining a single crystal of a quality sufficient for an XRD structural measurement (Figure S12).

**Figure 2:**
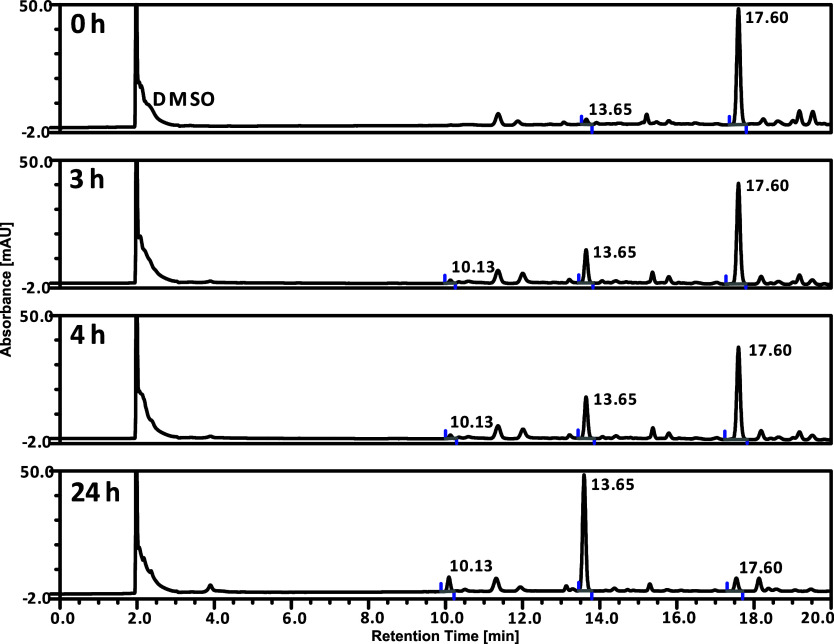
HPLC chromatograms of ***trans*-2** in 100% DMSO. The stability was measured after 0, 3, 4, and 24 h from sample preparation at ambient temperature. Peak identifications: 17.60 min—***trans*-2**; 13.65 min—***trans*-1**; 10.13 min—***cis*-1** (see Figure S10 in the Supporting Information for mass spectra).

Since the complex is poorly soluble in water (<3.7 μg/mL), it had to be introduced into the cell culture medium in DMSO (see the Cytotoxicity Tests section below). It is, however, well known that DMSO displaces ligands in Pt complexes, including cisplatin.^45^ Therefore, we decided to verify its stability in this solvent and 1% v/v DMSO in PBS. A 1.0 × 10^–4^ M solution of ***trans*-2** in 100% DMSO was stored in dark Eppendorf probes, to protect it from daylight, at ambient temperature for 0, 3, 4, and 24 h from sample preparation and its LC chromatograms were recorded (Figure 2).

**Figure 3:**
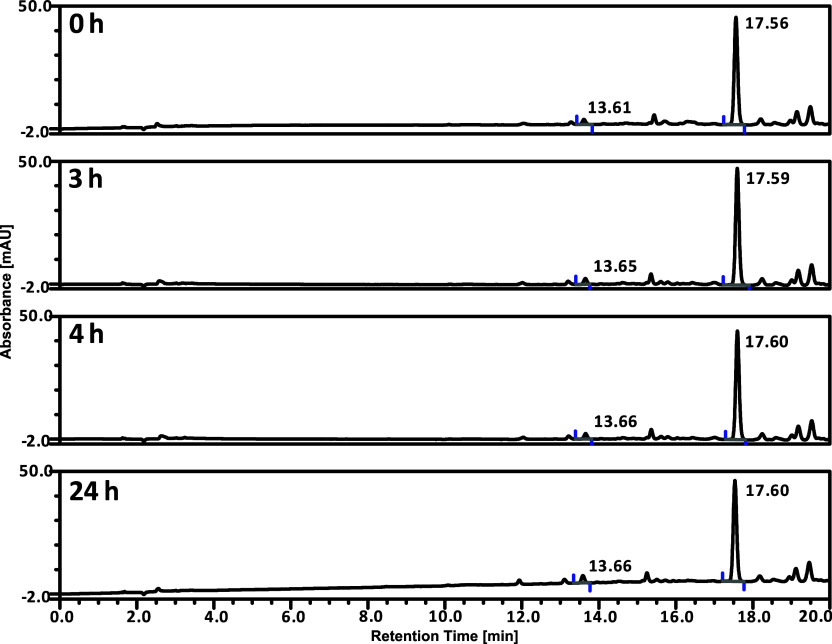
LC chromatograms of ***trans*-2** in 1% v/v DMSO in PBS. The stability was measured after 0, 3, 4, and 24 h from sample preparation at ambient temperature. Peak identification: 17.60 min—***trans*-2**; 13.65 min—***trans*-1**.

Figure 2 suggests that ***trans*-2** is unfortunately unstable in a 100% DMSO solution. Indeed, over time, the complex underwent degradation with the major formation of ***trans*-1**. Additionally, a small amount of the ***cis*-1** ligand was observed (see Figure 2, the signal at 10.13 min). After 24 h from solution preparation, ***trans*-2** decayed almost completely, despite protection from daylight. It was thus verified that in pure DMSO, the ***trans*-1** ligands in ***trans*-2** were substituted by the DMSO molecules. We therefore checked whether the complex was stable in PBS solution with 1% v/v DMSO. We found that the complex is completely stable under these conditions (Figure 3) which enabled the cytotoxicity tests to be performed.

### Photoswitching of **2** Studied with UV–Vis Spectroscopy

**Figure 4:**
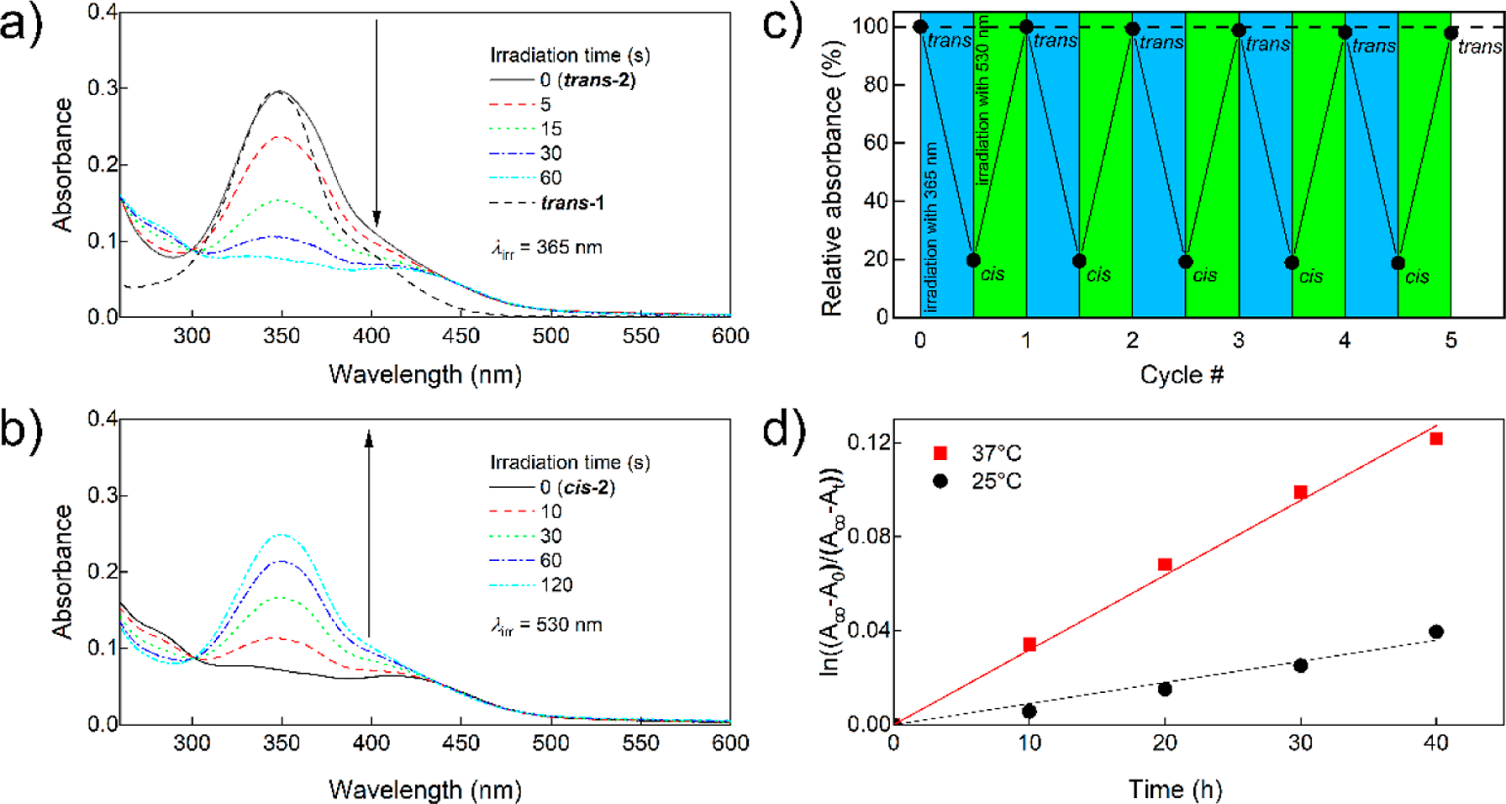
(a) UV–vis spectra of ***trans*-2** and (b) ***cis*-2** solution in 1% v/v DMSO in PBS (6.5 μg/mL, 8.6 μM) irradiated with 365 and 530 nm light, respectively. A spectrum of ***trans*-1** in PBS normalized to the same intensity at the 346 nm band maximum is given for comparison; (c) relative absorbance at 350 nm of **2** undergoing cyclic irradiation with 365 and 530 nm for up to 5 cycles (0.0125 mg/mL, 16.5 μM, 1% v/v DMSO). The irradiation times were 30 and 300 s, respectively, long enough to reach complete conversion; (d) first-order kinetic analysis of ***cis*-2** → ***trans*-2** thermal isomerization in pH 7.4 PBS containing 1% v/v DMSO.

UV–vis spectra of the ***trans*-2** and ***cis*-2** solutions in 1% v/v DMSO in PBS irradiated with 365 and 530 nm light are shown in Figure 4a,b, respectively.

They confirm that irradiation with 365 and 530 nm light leads to effective and reversible *trans–cis* and *cis*–*trans* photoisomerization, respectively, of the **1** ligands. The comparison of the spectrum of ***trans*-2** and that of ***trans*-1** normalized at the maximum of the π → π* absorption band (Figure 4a) reveals that at longer wavelengths, the absorption of the complex is relatively stronger than that of the respective photoisomer of **1**, resulting in a slight difference in color (yellow and orange, respectively) and, advantageously, much faster *cis*–*trans* photoisomerization of the complex compared to that of free ***trans*-1** at the same irradiation conditions. It should be pointed out that photoisomerizations of both **1** ligands in the complex are independent, so both photoisomerizations proceed through an intermediate complex structure in which both ligands have different configurations, i.e., *cis*-PtCl_2_(***trans*-1**)(***cis*-1**). However, since isolating this intermediate unstable product would be very challenging while its photochemical and biological properties are of secondary importance at the current stage of this study, we disregarded this point in further studies.

From the point of view of photoreversibility of the complex structure and consequently its biological properties, an important question is the photochemical stability of its **1** ligands and resistance to side photoreactions such as degradation, cyclization, dimerization, etc. during possible multiple back-and-forth *trans*–*cis* photoisomerizations. To address this problem, the complex was subjected to cyclic photoisomerizations, and the changes in the relative absorbance at the maximum of the ***trans*-2** absorption band (Figure 4c) were measured. The absorbance of ***trans*-2** after 5 cycles decreased by 2.3% which means that the linearly extrapolated cyclability,^10^*Z*_50_, of the complex **1** ligand is about 116 cycles. Resistance to fatigue of **1** as a ligand is comparable to that of other AAPs described in the literature^46^ and thus much higher than that required in any conceivable photopharmaceutical application.

Another important prerequisite for the photopharmacological application is that both photoisomers of the complex should also be thermally stable. To determine the kinetic parameters of thermal (dark) ***cis*-2** → ***trans*-2** isomerization, the UV–vis spectra of ***cis*-2** solutions were measured at 25 and 37 °C at pH 7.4. The absorption data fitted well to the assumed first-order kinetics (Figures 4d and S11). The values of the corresponding rate constants and half-lives obtained therefrom are given in Table 1.

**Table 1:** First-Order Rate Constants and Half-Lives for ***cis***-**2** → ***trans***-**2** Thermal Isomerization Obtained from the Data Shown in Figure 4D

	25 °C	37 °C
*k* [1/h]	9.01 × 10^–4^	3.18 × 10^–3^
τ_1/2_ [h/days]	770/32	218/9

The thermal half-life of the *cis* photoisomer of the complex under physiological conditions is 218 h (9 days). This half-life is similar to that of the free ***cis*-1** found in our previous work^40^ which means that the complexation of ***cis*-1** does not significantly influence its thermal stability. Thus, the thermal stability of both photoisomers of the complex is high enough to enable detection of possible differences in their biological properties (half-lives of the order of a few hours were considered as already long enough to determine biological properties of much less thermally stable photoisomers^36^).

## Results of Theoretical (Density Functional Theory) Calculations

**Figure 5:**
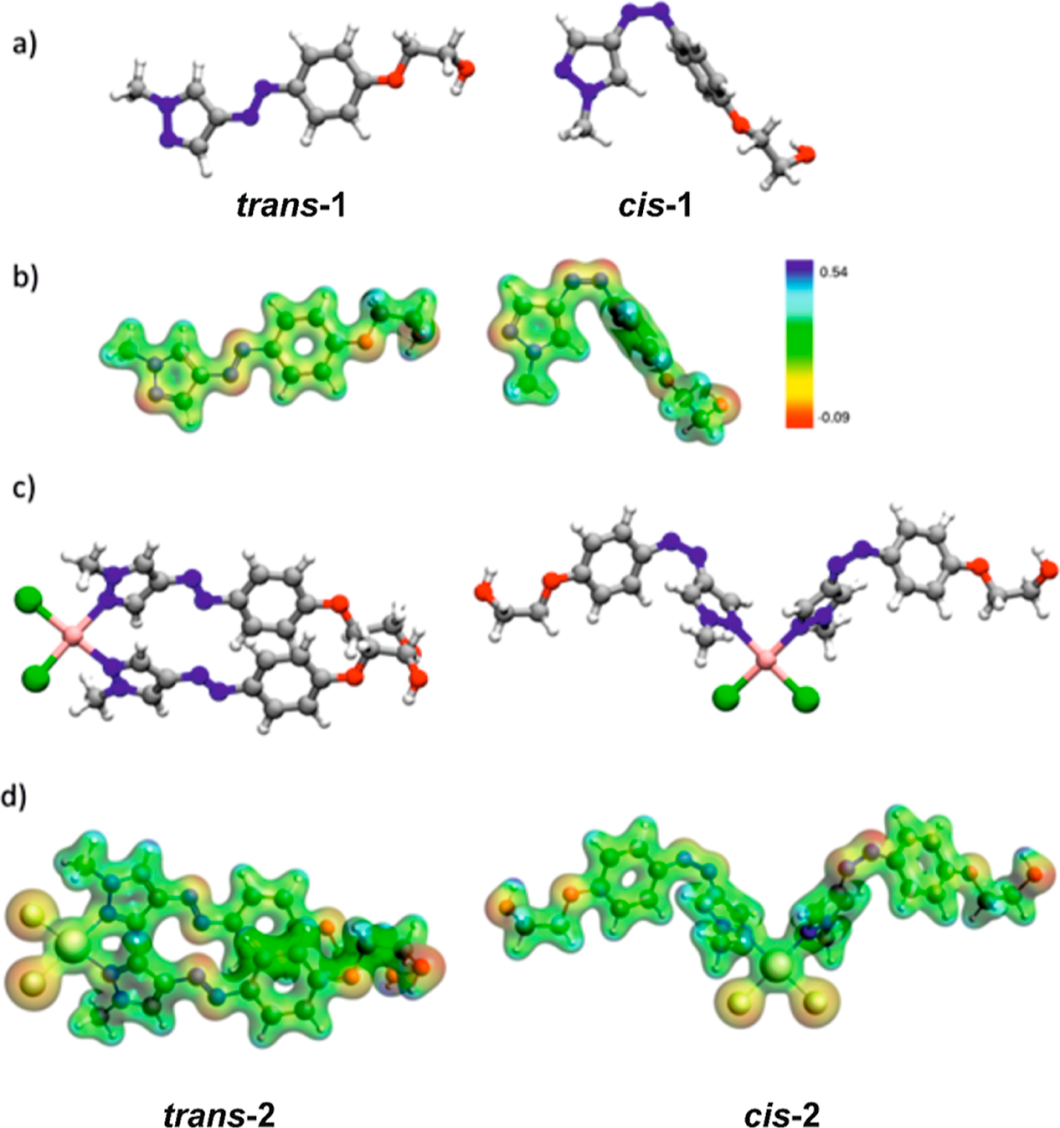
(a) Optimized geometries of ***trans*-1** and ***cis*-1** photoisomers, (b) molecular electrostatic potential (MEP) for ***trans*-1** and ***cis*-1** photoisomers (Δρ = 0.05), (c) optimized geometries of ***trans*-2** and ***cis*-2**, and (d) MEP for ***trans*-2** and ***cis*-2**.

To get some insight into the geometry of the ligand and the complex, electronic structure, photochemical properties, and stability of the complex, we have performed theoretical calculations with the density functional theory (DFT) method. In Figure 5, the optimized geometries of the models used in this study are shown. According to these calculations, the *trans* and *cis* isomers adopt significantly different geometries. Like in the case of *trans*-azobenzene, the aromatic scaffold of ***trans*-1** is planar, which is the effect of conjugation of π electrons from the diazo bond and both aromatic rings. In contrast to the similar geometries of *trans*-azobenzene and ***trans*-1**, those of ***cis*-1** and *cis*-azobenzene are quite different. Figure 5a shows that the pyrazole ring in ***cis*-1** is situated in the same plane as the diazo bond, almost perpendicular to the benzene moiety. This result is in agreement with the X-ray diffraction data obtained by Weston et al.^46^ It implies low oscillatory strengths of the π → π* transitions in ***cis*-1**, resulting from a decrease in the conjugation and the symmetry of the orbitals with π character. On the other hand, in *cis*-azobenzene, the two phenyl groups attached to the azo bonds are too hindered sterically to assume the relative perpendicular conformation. As a result, their mutual conformation is twisted. Selected structural parameters of **1** and azobenzene as a reference photoswitch are given in Section ST2 in the Supporting Information.

The above-described structural changes occurring upon photoisomerization cause the differences in the charge distribution between the **1** photoisomers. The MEP for both **1** photoisomers has been depicted in Figure 5b. The color contours show that the main nucleophilic areas within both **1** photoisomers are located on the nitrogen atoms of the azo-bond, the unmethylated nitrogen atom of pyrazole, and two oxygen atoms in the ether moiety. The differences in the spatial arrangement of these areas between *trans* and *cis* photoisomers together with the heterogenicity of the charge distribution in both photoisomers additionally result in the significant difference in their dipole moments which are equal to 8.01 and 5.03 D for ***trans*-1** and ***cis*-1**, respectively. The nucleophilic character of the pyrazole nitrogen atom is also an important chemical feature of **1** visible on the MEP contour map and is correlated with the ability of the moiety to coordinate the transition metals. The analysis of the photochemical properties of **1** and the comparison with those of azobenzene are given in the Supporting Information (Section ST2).

The optimization procedure of the complex geometry was performed for two systems in which the configuration of the **1** ligand was the same; therefore, the hypothetical situation when only one of the two **1** ligands undergoes photoisomerization was not taken into consideration (as decided above). Figure 5c presents optimized geometries of ***trans*-2** and ***cis*-2**. There are remarkable structural differences between both photoisomers of the complex, which are the cause of the differences in their biological activity described further. The planar aromatic scaffold appearing in the optimized unbound ***trans*-1** system is distorted in the complex and both ligands are no longer planar. However, it should be pointed out that *ab initio* MD results show that both conformers are stable at room temperature. The detailed analysis of structural parameters along with simulations has been discussed in the Supporting Information (Section ST3). MEP (shown in Figure 5d) qualitatively depicts the difference in the charge distributions of both photoisomers, resulting in the difference in their dipole moments. Similarly to the results obtained for unbound **1**, ***trans*-2** exhibits a notably larger dipole moment than ***cis*-2** (Table 2). The values of relative energies, *E*_rel_, and solvation energies, *E*_solv_, of *trans* and *cis* isomers are presented in Table 2. The ***trans*-2** system has a lower electronic energy, which is mostly due to the higher stability of the diazo bond configuration in the ligand. However, the *E*_solv_ of the *trans* photoisomer is less negative, indicating weaker solvation in comparison to that of the *cis* photoisomer. A higher absolute value of *E*_solv_, observed for ***cis*-2** indicates a greater stabilizing effect of solvation resulting from stronger spatial exposition of the **1** nucleophilic groups. This was also demonstrated in the MEP visualization (Figure 5d).

**Table 2:** Comparison of the Selected Properties (Dipole Moment, |**μ**_**D**_|, Relative Stability, ***E***_rel_, and Solvation Energy, ***E***_solv_) Obtained from the DFT Calculations for Both Complex Photoisomers

	***trans*-2**	***cis*-2**
|**μ**_**D**_| [***D***]	18.13	6.88
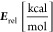	0	20.92
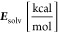	–46.09	–55.24

**Figure 6:**
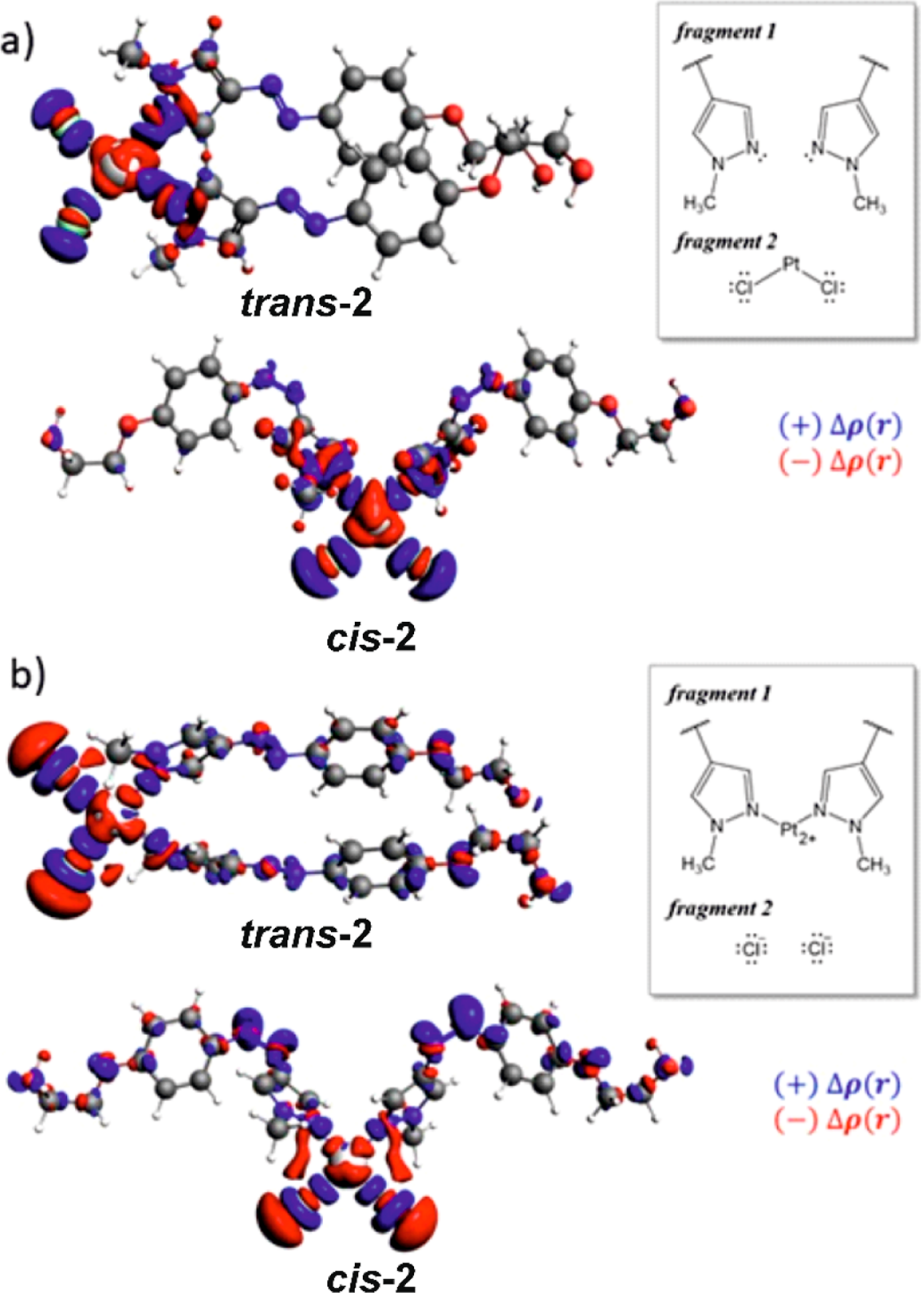
Differential density functions, Δρ, for the partitioning scheme depicting interaction between (a) **1** ligands and PtCl_2_ group and (b) chloride anions and Pt coordinated with **1** ligands (Δρ = 0.003).

The quantitative decomposition of the Pt–Cl and Pt–N bonds in the complex has been done via a two-ETS system scheme. ETS-1 provided the characteristics of the bonds between **1** ligands and the rest of the molecule, while ETS-2 corresponded to the interaction between chloride anions and platinum cation bonded to two **1** moieties (right panels in Figure 6).

The values of the energy decomposition terms, proposed by the Ziegler–Rauk energy decomposition analysis, are presented in Table 3. The interaction strengths, Δ*E*_bond_, between **1** ligands and the PtCl_2_ group for *trans* and *cis* complex photoisomers differ only slightly. The balance between steric and orbital interaction contributions elucidates the interaction character. In this partitioning, the electrostatic contribution is counterbalanced by the Pauli repulsion term, which is reflected in the small steric terms, similar for both photoisomers. The main stabilization comes from the orbital interaction contribution, Δ*E*_orb_. Qualitatively, the same results have been obtained for cisplatin. The donor character of this interaction and charge flow from the **1** ligand to the metal center is shown in Figure 6a. The second partitioning reflects the interactions between the Pt center and leaving group (Cl^–^) (Figure 6b) and expresses the tendency of the complex to undergo hydrolysis (substitution of chloride anions with water molecules), which is an important process activating Pt(II) compounds within cells. In ***trans*-2**, the interaction with the chloride leaving group is weaker than that in the ***cis*-2** photoisomer (−225.78 and −228.29 kcal/mol, respectively). This difference is, however, too small to explain the observed differences in the toxicity between the complex photoisomers (see the following sections). The difference between *cis* and *trans* photoisomers of the complex is again the largest in the electrostatic contribution, which is equal to −217 and −222 kcal/mol, for ***trans*-2** and ***cis*-2**, respectively. The energy terms obtained from the chosen energy decomposition approach were compared to the values obtained for cisplatin, indicating that in the complex, the **1** ligands are more strongly bound than the corresponding NH_3_ ligands in cisplatin. On the other hand, chloride ligands are less strongly bound in the complex than in cisplatin, suggesting that the complex has a stronger tendency for hydrolysis in the cells than cisplatin, which should contribute to its higher toxicity. The obtained values of the chloride ligand binding energy do not, however, explain the experimental observation that generally the *trans* and *cis* photoisomers are, respectively, more and less toxic than cisplatin (see the following sections).

**Table 3:** Calculated Values of ETS-Energy Decomposition Analysis Performed on the Considered Structures (All Values in kcal/mol per Ligand)

	Δ***E***_orb_	Δ***E***_elstat_	Δ***E***_Pauli_	Δ***E***_disp_	Δ***E***_bond_
		Δ***E***_ster_			
ETS-1					
**Cisplatin**	–47.97	–107.22	+117.65	–1.97	–39.51
		+10.43			
***trans***-**2**	–53.11	–102.43	+117.96	–6.74	–44.31
		+15.53			
***cis*-2**	–54.48	–108.89	+124.92	–6.88	–45.33
		+16.03			
**ETS-2**					
**Cisplatin**	–79.22	–269.88	+85.72	–1.31	–264.68
		–184.16			
***trans*-2**	–112.10	–217.29	+106.00	–2.39	–225.78
		–111.29			
***cis*-2**	–113.03	–221.82	+109.00	–2.44	–228.29
		–112.82			

The energy decomposition calculations performed explain the character of the considered metal–ligand interactions. First, the bonding between the PtCl_2_ moiety and the **1** ligands was shown to be the charge-transfer one, as supported by the values of the Δ*E*_orb_ term obtained and differential density visualizations. On the other hand, the interaction between the platinum cation attached to ligands with the chloride anions has an ionic character with strong polarization in the metal fragment. The Pt–Cl bonding in **2** is much weaker than that in cisplatin, which indicates a higher tendency for the Cl^–^ dissociation and a stronger electrophilic character of **2**. Additionally, the *trans*/*cis* conformation of the *N*-donating ligands does not impact the platinum center character; therefore, the photoisomerization should not significantly influence the reactivity of the metal complex center, as indeed found in the studies of the complex interaction with DNA (see below).

### *In Vitro* Cytotoxicity of the Complex

The ultimate goal of the studies was to test whether the desirable significant difference exists between the toxicities of both photoisomers of the complex and how they compare to that of cisplatin. For this purpose, the cytotoxicity of both photoisomers of the complex was extensively tested in a few cell lines, murine and human, both cancer and normal ones, in a culture medium with and without the addition of 10% FBS (referred to later as serum- and serum-free conditions, respectively). The selected cell lines originated from organs whose cancers may be treated with cisplatin, i.e., breast cancer, melanoma, and ovary cancer. Only the NMuMG murine mammary normal cells could not be cultured in a completely serum-free medium, and to grow these cells, the addition of 1% FBS was necessary (referred to later as near-serum-free conditions). Before the cytotoxicity tests were started, it was not possible to predict which of the photoisomers would be more toxic. Moreover, literature data indicate that it is difficult to anticipate which photoisomer will be more active, even having some prior theoretical modeling data favoring one of the photoisomers.^36^ The metabolism of the complex upon uptake by the cells is unknown, and one should consider some release of the **1** ligand from the complex, as found from the ^1^H NMR study of the complex photoisomerization (Figure S5). Therefore, to rule out the possibility that the cytotoxicity of the complex may be due to the cytotoxicity of the released **1** ligand, the latter was determined in the B16-F10 murine melanoma, 4T1 murine mammary cancer, and NMuMG murine mammary cell lines, both in serum and serum-free conditions, after both 24 and 48 h of exposure (Figures S13–S15). Both photoisomers of **1** ligand were found nontoxic in all tested cell lines in serum conditions for up to 48 h. In serum-free conditions, ***cis*-1** showed cytotoxicity that grew with time and was significantly different from those of the control and ***trans*-1**. However, in none of the cell lines, the cytotoxicity of the ***cis*-1** photoisomer exceeded 50%.

**Figure 7:**
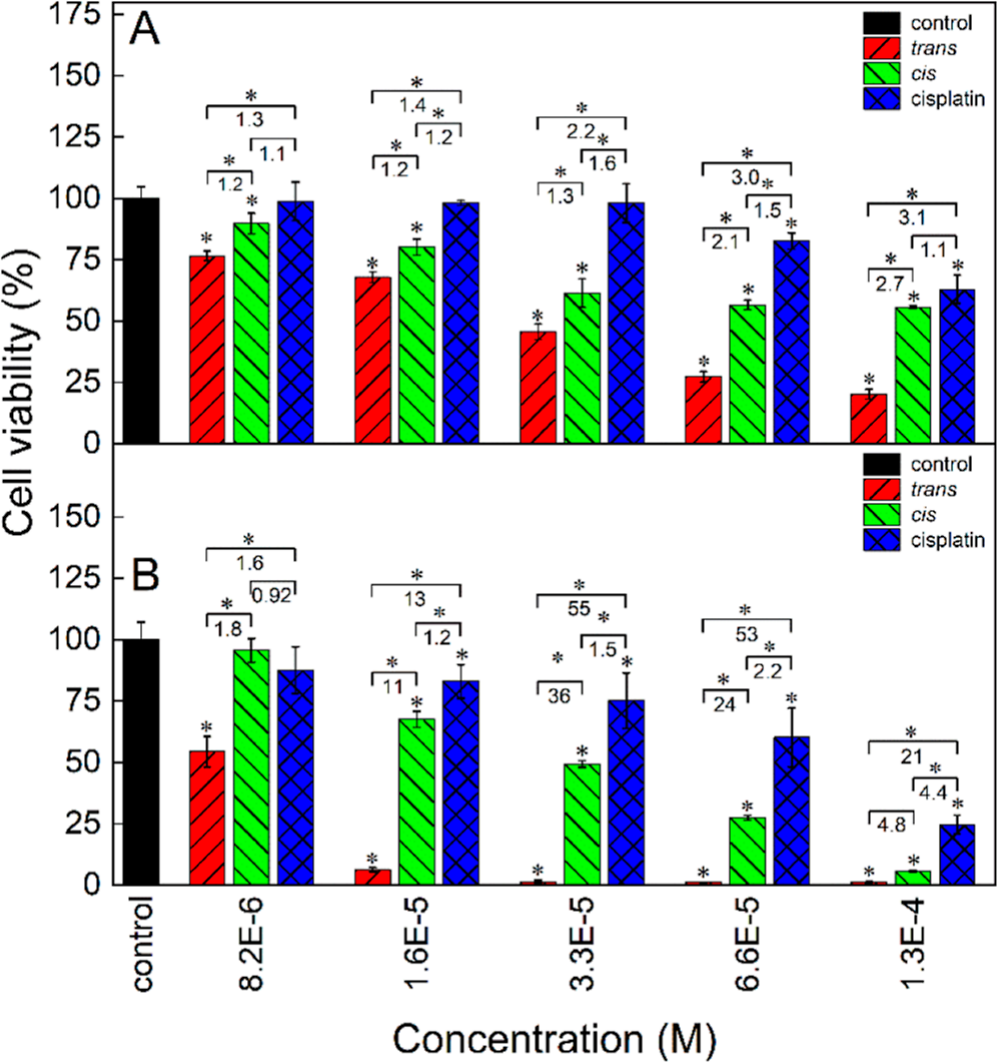
Cytotoxicity of ***trans*-2**, ***cis*-2**, and cisplatin in the B16-F10 murine melanoma cell line after 24 (A) and 48 h (B) of cell culture determined with the MTT test. The culture medium (DMEM–high glucose) was supplemented with 10% FBS. The numbers denote the *cis*/*trans*, cisplatin/*cis,* and cisplatin/*trans* viability ratios at respective concentrations. Statistically significant differences are indicated with asterisks above the bars (difference from the control) and above the brackets (difference between the photoisomers and between cisplatin and either of the photoisomers), *p < 0.05.

The expected difference between the toxicities of complex photoisomers was indeed found in all studied cell lines, human and murine, normal and cancer ones, after both tested culture times (24 and 48 h) and irrespective of the presence of serum in the culture medium. Namely, it was the *trans* photoisomer of the complex that was always more toxic than the *cis* photoisomer and, importantly, also more toxic than cisplatin. In the B16-F10 murine melanoma cell line after 24 h of exposition (Figure 7A), the cytotoxicity of the *trans* photoisomer was significantly higher than that of the *cis* photoisomer at all concentrations and the ratio of the cell viabilities for the *trans* and *cis* photoisomers (hereafter referred to as the *cis*/*trans* viability ratio) grew with increasing concentration, reaching 2.7 at 1.3 × 10^–4^ M.

Both photoisomers were more toxic than cisplatin; the *trans* photoisomer was up to 3.1 times more toxic, while the *cis* photoisomer was up to 1.6 times. After 48 h of cell culture (Figure 7B), the differences among the cytotoxicities of the three species were much more pronounced than those after 24 h. At 3.3 × 10^–5^ M, the *cis*/*trans* and cisplatin/*trans* viability ratios reached maximum values as high as 36 and 55, respectively. The cisplatin/*cis* cytotoxicity ratio grew steadily with the concentration reaching 4.4 at 1.3 × 10^–4^ M.

In serum-free conditions (Figure S16), the relationships among the cytotoxicities of the three species were qualitatively similar; however, after 48 h, the cytotoxicity of the *trans* complex photoisomer was very high already at the lowest concentration of 8.2 × 10^–6^ M, much higher (13% viability) than in serum conditions (54% viability).

The opposite relationship between the cytotoxicities of both forms of **2** (*trans* photoisomer being always more toxic than the *cis* one) and **1** (the *cis* photoisomer more toxic than the *trans* one and significantly toxic only at longer culture time, at serum-free conditions, and at highest concentrations, Figures S13–S15) indicates that the cytotoxicity observed in the cells supplemented with the complex should be ascribed to the presence of the complex itself and not to the **1** ligand released due to irradiation.

**Figure 8:**
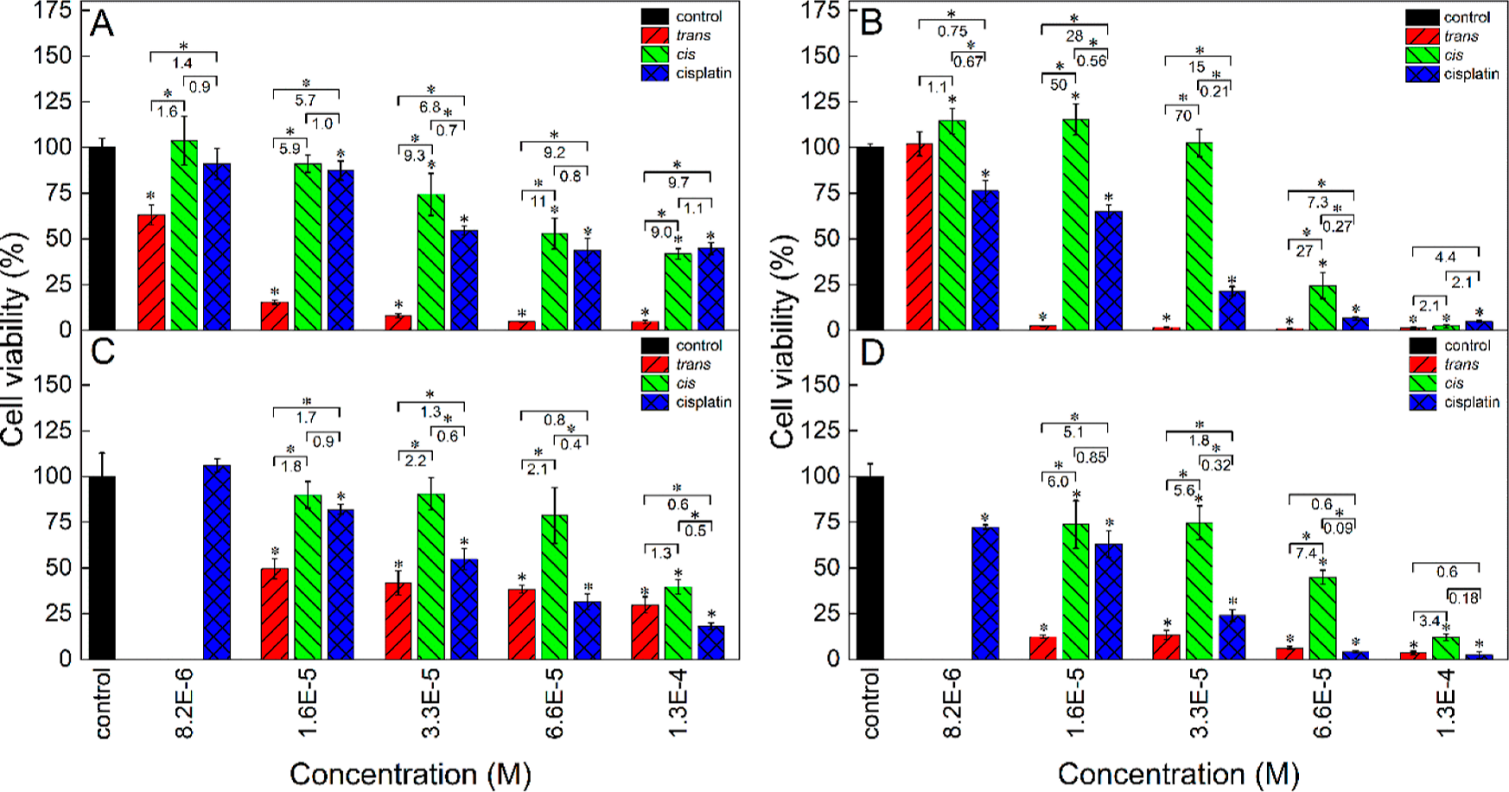
Cytotoxicity of ***trans*-2**, ***cis*-2**, and cisplatin in the 4T1 murine mammary cancer (A,B) and normal NMuMG (C,D) cell line after 24 (A,C) and 48 h (B,D) of cell culture determined with the MTT test. The culture medium (DMEM—high glucose) was supplemented with 10% FBS. The numbers denote the *cis*/*trans*, cisplatin/*cis*, and cisplatin/*trans* viability ratios at respective concentrations. Statistically significant differences are indicated with asterisks above the bars (difference from the control) and above the brackets (difference between the photoisomers and between cisplatin and either of the photoisomers), *p < 0.05.

The analogous cytotoxicity tests were performed in the 4T1 murine mammary cancer cells after 24 and 48 h of cell culture, and the cytotoxicities of both photoisomers were again compared to that of cisplatin as a reference drug (Figure 8A,B).

The *cis*/*trans* viability ratios for 24 h culture were even higher than in the B16-F10 cell line and ranged from 1.6 to 11 (Figure 8A). The cytotoxicity of the *cis* photoisomer was very similar to that of cisplatin with a cisplatin/*cis* viability ratio ranging from 0.7 to 1.1. The *trans* photoisomer was generally much more toxic than cisplatin, and the cisplatin/*trans* viability ratio ranged from 1.4 to almost 10. The differences between the toxicities of both photoisomers and between cisplatin and the *trans* photoisomer dramatically increased upon prolonging the exposition time to 48 h (Figure 8B). The *cis*/*trans* and cisplatin/*trans* viability ratios reached very high values of 70 at 3.3 × 10^–5^ and 28 at 1.6 × 10^–5^ M, respectively. After 48 h, the *trans* photoisomer killed almost all cancer cells, except for its lowest concentration (8.2 × 10^–6^ M), while the *cis* photoisomer was nontoxic at concentrations up to 3.3 × 10^–5^ M. Thus, there was a concentration window (between 8.2 × 10^–6^ and 6.6 × 10^–5^ M) where the *trans* photoisomer was toxic to almost 100% cells, while the *cis* photoisomer was completely nontoxic to these cancer cells. Beneficially, after 48 h of exposition, the cytotoxicity of cisplatin was found to be intermediate between the cytotoxicities of both complex photoisomers at most of the concentrations (except for the concentration of 8.2 × 10^–6^ M at which it was higher than the cytotoxicity of the *trans* photoisomer).

In serum-free conditions after 24 h, the results for 4T1 cells were qualitatively comparable to those obtained for 24 h culture time in serum conditions (compare Figures S17A and 8A). After 24 h, the cisplatin/*cis* cytotoxicity ratios ranged from 1.0 to 1.3, indicating that the cytotoxicity of the *cis* photoisomer was equal to or only slightly higher than that of cisplatin. The respective cisplatin/*trans* ratios ranged from 1.4 to 16, denoting much higher toxicity of the *trans* photoisomer than that of cisplatin. The ranges of *cis*/*trans* viability ratios in serum and serum-free conditions were similar, indicating much higher cytotoxicity of the *trans* photoisomer in both conditions.

For 48 h, the cytotoxicity of cisplatin in serum-free conditions was similar to that of the *cis* photoisomer (Figure S17B), in contrast to serum conditions (Figure 8B) at which it was higher. Like in the serum conditions, there was a concentration window at which the *trans* photoisomer killed almost all the cells and the *cis* photoisomer was nontoxic, although it was narrower than in serum conditions (between 8.2 × 10^–6^ and 3.3 × 10^–5^ M).

Cytotoxicity of cisplatin and both forms of the complex was also tested in the normal murine mammary cell line (NMuMG) both in serum (Figure 8C,D) and in near-serum-free conditions (with the addition of 1% FBS, Figure S17C,D). In the serum conditions, the toxicity of the *trans* photoisomer after 24 h was, like in previously described cell lines, higher than that of the *cis* photoisomer and the *cis*/*trans* viability ratios ranged from 1.3 to 2.2 (Figure 8C), so the cytotoxicities of both photoisomers did not differ as much as in the case of corresponding 4T1 cancer cells at the same culture conditions (Figure 8A). After 48 h of the cell culture, the *cis*/*trans* viability ratios for NMuMG cells increased (Figure 8D) compared to those after 24 h and ranged from 3.4 to 7.4, reflecting a greater difference in the toxicities of both complex photoisomers, but these ratios were still much lower than the respective ratios for the 48 h culture of the corresponding cancer cells (4T1, Figure 8B).

In near-serum-free conditions after 24 h of NMuMG cell culture, the toxicity of cisplatin was comparable to that of the *cis* photoisomer of the complex with cisplatin/*cis* viability ratios ranging from 0.68 to 1.0 (Figure S17C). The *trans* photoisomer was moderately toxic at the whole range of concentrations and was only up to 2.0 times more toxic than the *cis* photoisomer. After 48 h of NMuMG cell culture (Figure S17D), the *trans* photoisomer was very toxic at the whole range of concentrations, while the *cis* photoisomer was nontoxic up to 3.3 × 10^–5^ M, and at higher concentrations, it was very toxic. The toxicity of cisplatin did not change significantly with culture time except for the highest concentrations.

By comparing the respective data for cancer (4T1) and normal (NMuMG) murine breast cells, an important conclusion may be reached that the *trans* photoisomer was always more toxic for cancer than for normal cells, for both 24 and 48 h incubation times and for both serum- and serum-free conditions (compare red bars in Figure 8A,C, in Figure 8B,D, in Figures S17A,C, and in Figures S17B,D).

Preliminary measurements of the cytotoxicity of both photoisomers were also done in the A2780 human ovary carcinoma cell line (Figure S18) after 24 h of culture under serum-free conditions. Also in this case, the *trans* photoisomer was more toxic than the *cis* one and the *cis*/*trans* viability ratios were quite high reaching about 9.

The fact that the *trans* photoisomer was found to be always more toxic has another important implication. Since the *cis*–*trans* thermal isomerization of AAPs is strongly accelerated by acids (by up to 5 orders of magnitude^47^), it may be also expected that the *cis*–*trans* dark isomerization of the complex *in vivo* will be faster in the extracellular matrix of cancerous than of normal tissue because of the Warburg effect.^48^ Thus, the half-life of the *cis* photoisomer in cancer tissue may be shorter than 9 days. The potential consequences of this fact are yet to be determined in *in vivo* studies.

### Cellular Accumulation of the Complex

**Figure 9:**
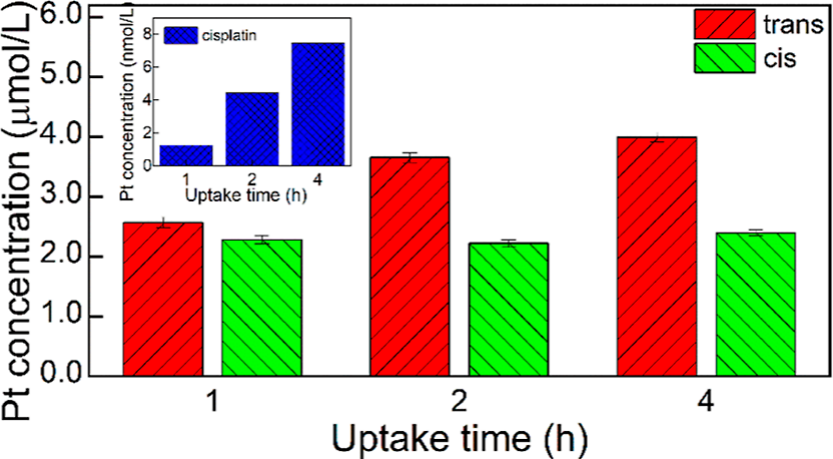
Pt concentration in the lysates of 4T1 cells incubated for various times with ***trans*-2**, ***cis*-2**, and cisplatin. Since the Pt concentration in the lysates of cells exposed to cisplatin was 3 orders of magnitude lower than in cells exposed to either of the complex photoisomers, the cisplatin bars would be not visible on the common Y-scale; therefore, the data for cisplatin are shown in the inset.

The results of the above experiments could not answer the question of whether the observed significant difference in the cytotoxicity of both complex photoisomers could be due to a higher uptake of the *trans* photoisomer (assuming their similar toxicity), higher intrinsic toxicity of the *trans* photoisomer, or both factors. To resolve this question, we have measured the uptake of both complex photoisomers and cisplatin after exposing the 4T1 cells to these compounds for various times. The results are shown in Figure 9.

It was found that the uptake of both photoisomers is much stronger than that of cisplatin. This difference is particularly high for 1 h uptake time (Pt concentrations are 2.0 × 10^3^ and 1.8 × 10^3^ higher for ***trans*-2** and ***cis*-2**, respectively, than for cisplatin) and decreases with uptake time (down to 5.3 ×10^2^ and 3.2 ×10^2^, respectively, for a 4 h uptake time). Also, the uptake of the *trans* photoisomer grew with incubation time while that of the *cis* one was completed within 1 h and did not change within 4 h. Importantly, the uptake of the *trans* photoisomer was higher after 1 h of incubation (by about 12%) and this difference increased with the incubation time, reaching 67% after 4 h. Thus, a significant and time-dependent difference in the uptake of both photoisomers was found, which probably contributed to the observed higher toxicity of the *trans* photoisomer.

### Photoswitching of the Cytotoxicity of the Complex upon Uptake by the 4T1 Cells

Our next goal was to investigate if there are also differences in the intrinsic toxicity between the photoisomers, not related to the differences in their uptake. To answer this question, the 4T1 cells were incubated for different times with *trans* and *cis* photoisomers. Next, the medium was replaced with a clean one, the cells were irradiated in the incubator with 365 and 530 nm light, respectively, to induce *trans*–*cis* and *cis*–*trans* photoisomerization, respectively, and the cells were cultured for 48 h. Thus, the light-induced changes in the toxicity of only the complex that was internalized by the cells could be studied.

**Figure 10:**
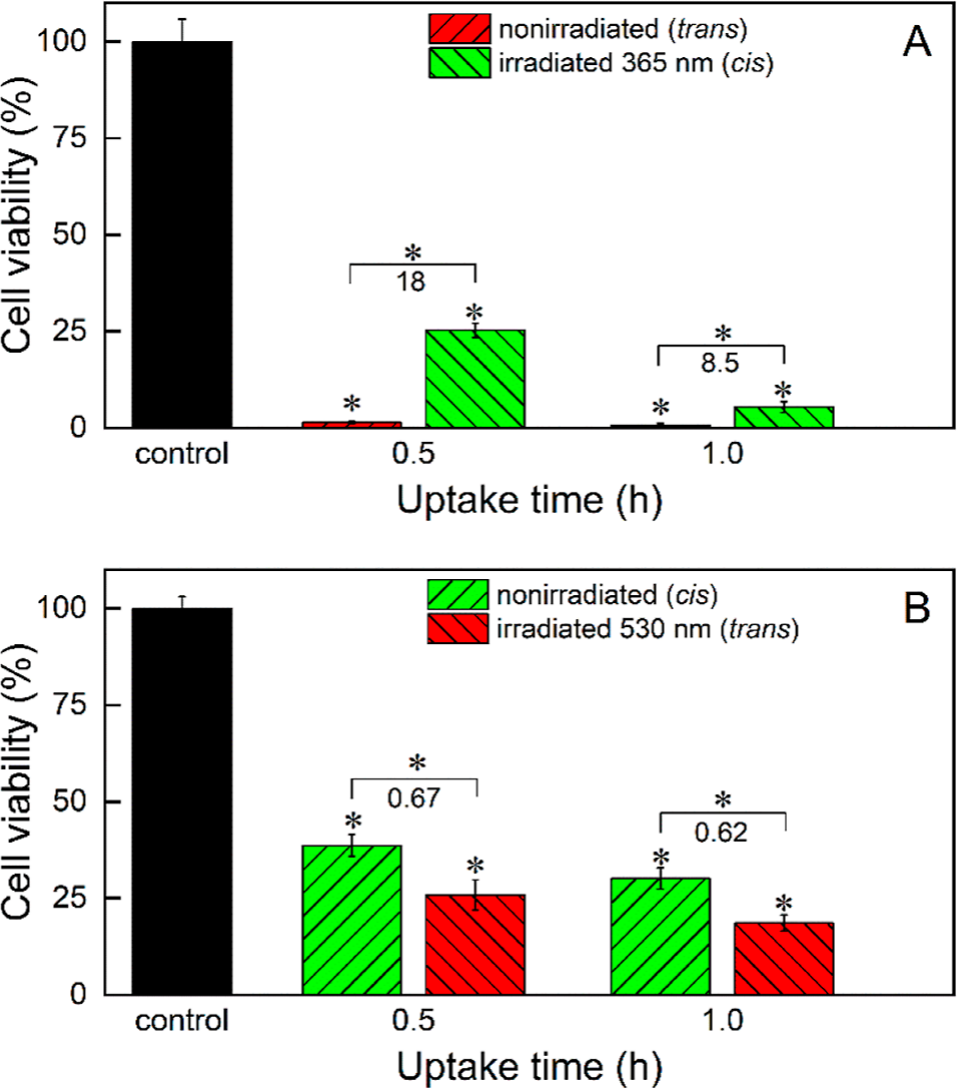
Cytotoxicity of (a) ***trans*-2** and (b) ***cis*-2** nonirradiated and irradiated upon internalization by the 4T1 cells after 48 h of culture in DMEM–high glucose determined with the MTT test. The cells were irradiated directly after complex uptake for 5 and 200 s to convert the *trans* photoisomer into the *cis* one and *vice versa*, respectively. The numbers denote the irradiated/nonirradiated viability ratios at a respective uptake time. Statistically significant differences are indicated with asterisks above the bars (difference from the control) and above the brackets (difference between nonirradiated and irradiated photoisomers), *p < 0.05.

The *trans–cis* photoisomerization of the *trans* photoisomer resulted in a significant decrease in toxicity (18 and 8.5 times for the uptake time of both 0.5 and 1 h, respectively, Figure 10A). Conversely, the irradiation of the cells incubated with the *cis* photoisomer with 530 nm followed by its *cis*–*trans* photoisomerization resulted in a decrease in the toxicity (by 33 and 38% for the uptake time of 0.5 and 1 h, respectively, Figure 10B). It was also verified that doubling the irradiation time of the *trans* and *cis* photoisomers (to 10 and 400 s, respectively) did not statistically change the results (data not shown), which additionally excluded the influence of the toxicity of light on the results and confirmed complete photoisomerization of both complex forms within the cells. The data obtained indicate unambiguously that the *trans* photoisomer is intrinsically more toxic than the *cis* photoisomer and that it is possible to both activate and deactivate the complex with light. Also, we found that the effect of decreasing the complex toxicity by its *trans–cis* photoisomerization is much stronger than the effect of increasing the complex toxicity through its *cis*–*trans* photoisomerization in the cells. The smaller toxicity change upon irradiation for the *cis* photoisomer can be at least in part due to its lower uptake (Figure 9) and in part due to the photodissociation of the complex (Figure S5).

### Mechanism of Cellular Death Induced by the Complex

One of the most important questions that should be raised upon finding high toxicity of the complex concerns the mode of cellular death it induced. Cisplatin was reported to trigger mostly apoptosis,^49−51^ but it can also induce necrosis,^52^ or both.^53^ On the other hand, the pyrazole complexes of Pt(II), which are structurally related to **2**, were found to cause apoptosis.^54^ The annexin V binding test carried out using 4T1 cells indicated that both **2** photoisomers caused necrosis of the 4T1 cells (Figure S19), with the number of necrotic cells being higher for the *trans* photoisomer. Since the type of cell death induced by Pt complexes may vary for different cancer cells and even within the heterogeneous collection of the cells within a tumor, further studies on the cell death mechanism in different cancer cells are required.

### Interaction of the Complex and Cisplatin with DNA and Albumin Studied Using Circular Dichroism Spectroscopy

**Figure 11:**
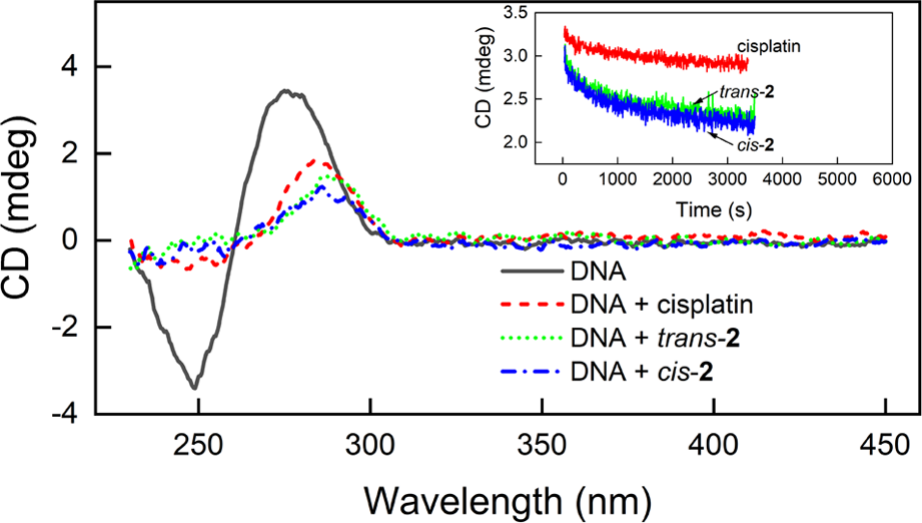
CD spectra of free calf thymus DNA (230 μM base pair) before and after its incubation with both complex photoisomers (2 h of incubation) and with cisplatin (20 h of incubation) as a positive reference. The concentration of all platinum compounds was 250 μM. Inset: the interaction kinetics tracked at 275 nm. All measurements were performed in 10 mM sodium phosphate buffer pH 7.4 at 25 °C.

The impact of ***trans*-2** and ***cis*-2** on naked DNA from the calf thymus was analyzed using circular dichroism (CD) spectroscopy and compared to that of cisplatin. Adding the studied compounds to DNA causes significant changes in its CD spectra, as shown in Figure 11. The initial DNA spectrum is characteristic of the B form of the double helix with a minimum in the 250 nm band and a maximum in the 270 nm band. In the presence of both complex photoisomers and cisplatin, the minimum disappeared while the maximum was reduced in amplitude and red-shifted. These changes indicate that both the complex and cisplatin induce changes in the DNA structure,^55^ however, for the complex, they are more pronounced. On the other hand, in the range above 320 nm, in which **1** ligands absorb, no induced CD signal was observed; thus, no chirality was induced in the structure of the ligand. This may indicate that the part of the complex molecule involved in the interaction with DNA is that opposite to the **1** ligands, i.e., chloride (or more probably water) ligands. It may therefore be speculated that the nature of the interaction with DNA is the same for both the complex and cisplatin. However, there were significant differences in the kinetics of the interaction between the complex and cisplatin, as shown in the inset in Figure 11. For ***trans*-2** and ***cis*-2**, the characteristic spectral changes were obtained already after 2 h, while for cisplatin after 20 h of incubation of the sample, the changes in the CD spectrum of the DNA were still less pronounced than for both complex photoisomers. On the other hand, the rate of the changes in the DNA structure caused by both complex photoisomers is very similar. Thus, the complex favorably shows much stronger and faster interaction with naked DNA than cisplatin. The higher rate of this interaction for the complex can be contrasted with another cisplatin analogue, AMD473, containing a 2-methylpyridine ligand, which was bound by DNA at a much slower rate than cisplatin.^39^ This difference is probably related to the difference in the electronic effects, thermal stability, and release of the 1*N*-methylpyrazole and 2-methylpyridine ligands under biological conditions.

### Interaction of the Complex with Bovine Serum Albumin

**Figure 12:**
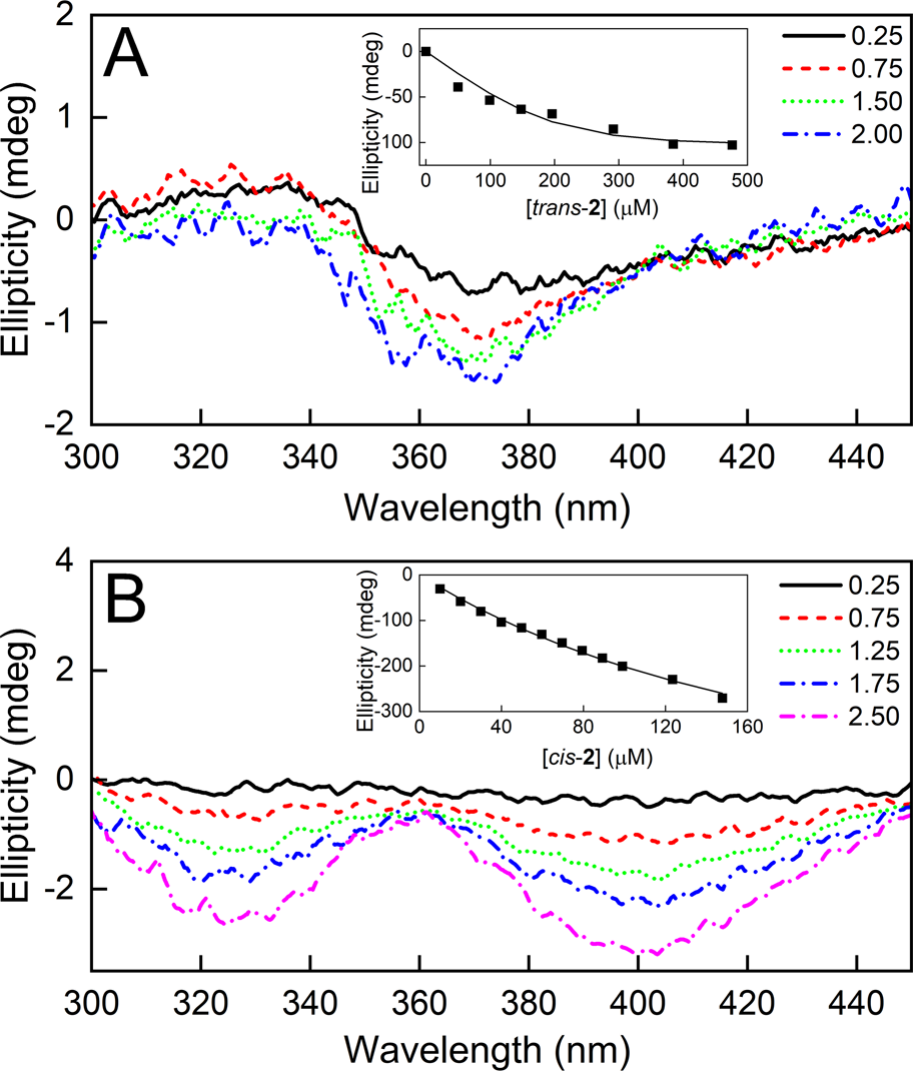
CD spectra of BSA complexes with ***trans*-2** (A) and ***cis*-2** (B). Measurements were done in PBS pH 7.4 at 25 °C. Numbers shown in the legends indicate the molar excess of **2** relative to BSA. Insets: exemplary dependence of the CD signal intensity relative to **2** concentration. Solid lines represent the best fit of the measurement data to the single-site binding model.

Binding to the plasma proteins, mainly albumin, is an important aspect of cisplatin activity. It was thus important to verify whether **2** is also bound by this protein, as expected, and whether there are some differences in the binding of both photoisomers. The binding of ***trans*-2** and ***cis*-2** to BSA was monitored by measuring the CD spectra. The results are shown in Figure 12. Upon the addition of ***trans*-2** to the BSA solution, the minimum centered at 370 nm on the spectrum appeared. In the case of ***cis*-2**, two minima centered at 330 and 400 nm appeared. The amplitudes of these extremes depended on the concentration of the added compounds.

In the selected spectral range, BSA does not absorb light and the complexes do not have CD spectra (data not shown). Thus, the appearance of a CD signal indicates the interaction of both complex photoisomers with BSA. The CD signal obtained is of low intensity but sufficiently strong to allow affinity analysis. Global analysis of three measurements performed for different concentrations of reactants using a single-binding-site model yields satisfactory fits (examples of single titration with the fit are shown in the insets in Figure 12). According to this model, BSA binds ***trans*-2** and ***cis*-2** with *K*_D_ values equal to 30 ± 15 and 184 ± 25 μM, respectively. It is generally assumed that the binding of cisplatin to serum proteins is irreversible; therefore, the relevance of the *K*_D_ value obtained for the complex may seem questionable at first glance considering the structural analogy of the coordination center of both compounds. However, one should take into account that the irreversible binding of cisplatin to proteins occurs within a time scale of several hours, while for the first few hours, both irreversible and reversible binding mechanisms are expected.^56^ CD measurements indicate that the binding of the complex occurs within the time scale of seconds, and importantly, the complex binds to BSA through the ligand. Thus, the binding of the complex to BSA in the second time scale can be considered reversible. The fact that the *trans* photoisomer is more strongly bound indicates that, first, *trans–cis* photoisomerization of the *trans* photoisomer bound to BSA should result in its partial release from the complex with BSA (and probably also from the complex with HSA) and, second, the difference in binding of both photoisomers to BSA present in FBS added to the culture medium cannot be the cause of the difference in their cytotoxicity since the more strongly bound *trans* photoisomer is also more toxic for both cancer and normal cells cultured in the presence of FBS (in serum conditions) than the *cis* photoisomer. Also, there is no clear difference in the toxicity of the *trans* photoisomer between serum-free and serum conditions.

### Influence of the Complex on the Glutathione and Cysteine Level

The level of cellular thiols, of which GSH is the most abundant, is an important factor influencing the anticancer activity of platinum-based drugs since they are deactivated by these compounds. The change in the level of cysteine (CYS) and both reduced (GSH) and oxidized (GSSG) forms of glutathione induced by the complex was studied in 4T1 cells. The data obtained are shown in Table 4.

**Table 4:** Levels of Reduced and Oxidized Glutathione and Cysteine (nmol/mg Protein) in the 4T1 Cells Treated with the Complex

	CYS	GSH	GSSG	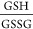
control	3.0 ± 0.6	65.0 ± 5.3	0.94 ± 0.2	70.8 ± 11.9
***cis*-2**	3.7 ± 0.5	38.3 ± 2.3	0.32 ± 0.1	120.1 ± 16.5
***trans*-2**	7.3 ± 0.8	29.0 ± 3.0	0.59 ± 0.02	52.9 ± 6.6

A decrease in GSH (greater for the *trans* complex) and GSSG (greater for the *cis* complex) levels was significant for both photoisomers of the complex. However, the level of GSH was reduced more by the more toxic *trans* photoisomer of the complex (down to about half of its control level). It is an important observation since, on the one hand, the reaction of cisplatin with GSH is one of the mechanisms of its deactivation, and, second, the concentration of GSH in cisplatin-resistant cancer cells is often increased compared to nonresistant ones.^57^ It is known that GSH actively participates in the metabolism of xenobiotics, mainly by forming conjugates with electrophilic metabolites and scavenging free radicals formed in the cells.^58^ The cause of this decrease in GSH is unknown, but it may likely be the result of, for example, a direct reaction of GSH with the complex. It may also be the result of the detoxification of reactive metabolites formed in cells during the metabolism of the tested compounds or the sum of the above-mentioned factors. As the cysteine residue of GSH can be readily oxidized nonenzymatically to disulfide (GSSG), the level of the latter compound was also determined, and it was found that the level of GSSG was decreased more profoundly by the *cis* photoisomer of the complex (down to about one-third of its control level). The level of reduced GSH is higher after the addition of the *cis* photoisomer, and the level of GSSG is lower compared to the test group treated with the *trans* photoisomer. Consequently, the ratio of GSH/GSSG, the major redox couple that determines the antioxidative capacity of the cells^59^ which is high when the redox state in cells is maintained, is more favorable for the *cis* complex photoisomer than for the *trans* one. The GSH/GSSG ratio of the *trans* photoisomer is the lowest among those analyzed, which may be partially responsible for its higher toxicity. The lowest GSH/GSSG ratio reflects poorer cellular conditions for maintaining the redox state; the equilibrium is shifted toward the formation of GSSG, which may contribute to the development of oxidative stress in the cells. On the other hand, in cells treated with the *trans* photoisomer, the CYS level significantly increased compared to the control, while for the *cis* photoisomer, its increase was not considerable. The bioavailability of CYS is a limiting factor for the synthesis of new GSH molecules. However, the 2-fold increase in CYS level in the *trans* photoisomer group compared to the control and *cis* photoisomer groups does not imply that the excess CYS is used for GSH synthesis (and probably is not used as the GSH level in the *trans* photoisomer group is the lowest). In this case, CYS is probably diverted to other metabolic pathways or is a substrate for the formation of compounds such as metallothioneins, thioredoxin, or coenzyme A.

## Conclusions

A photoactive cisplatin analogue was synthesized which is a square planar Pt(II) complex with two chloride ligands and two photoswitchable arylazopyrazole (AAP) ligands in the *cis* configuration in the coordination center. The complex efficiently undergoes *trans–cis* and *cis*–*trans* photoisomerizations when irradiated with 365 and 530 nm light, respectively. Photoisomerization is accompanied by significant changes in the molecule geometry and, consequently, in complex toxicity versus several cell lines, both normal and cancer ones (B16-F10, 4T1, NMuMG, and A2780). The *trans* photoisomer was found to be always more toxic than the *cis* one and also always more toxic than cisplatin. It was also more toxic to cancer 4T1 cells than to normal cells of the murine mammary gland. The uptake of the *trans* photoisomer by 4T1 cells was stronger than that of the *cis* photoisomer, while both photoisomers were taken up much more strongly than cisplatin. The irradiation with 365 nm light of the *trans* photoisomer internalized by the 4T1 cells resulted in a decrease in the toxicity of the complex, while the irradiation of the internalized *cis* photoisomer with 530 nm light resulted in an increased toxicity. The complex caused cellular death in the necrotic mechanism.

## Experimental Section

### Reagents and Materials

K_2_PtCl_4_ (98%, Angene), cisplatin (99%, AmBeed), acetonitrile (POCH), formic acid (Sigma-Aldrich), DMSO (Sigma-Aldrich), pH 7.4 PBS tablets (Sigma-Aldrich), BSA (Acros), MTT reagent (3[4,5-dimethylthiazol-2-yl]-2,5-diphenyltetrazolium bromide, AmBeed), Dulbecco’s modified Eagle’s medium—high glucose (DMEM, Sigma-Aldrich), RPMI-1640 cell culture medium (Sigma-Aldrich), fetal bovine serum (FBS, Sigma-Aldrich), l-glutamine (99.0–101.0%, Sigma-Aldrich), insulin (human, Sigma-Aldrich), and trypsin–EDTA solution (Sigma-Aldrich) were the reagents used.

### Synthesis of ***trans*-2**

Synthesis of (*E*)-2-(4-((1-methyl-1*H*-pyrazol-4-yl)diazenyl)phenoxy)ethan-1-ol (***trans*-1**) was performed according to the procedure described in our previous paper.^40^ The synthesis of ***trans*-2** (Figure S1) was performed based on modified literature procedures.^29−31,41^ Briefly, 2.31 g (9.35 mmol) of ***trans*-1** was suspended in 300 mL of methanol, added to the solution of 1.96 g (4.72 mmol) K_2_PtCl_4_ in 150 mL of water, and mixed at room temperature for 48 h. Then, half of the volume of methanol was removed under reduced pressure. The precipitated product was centrifuged (5 min, 10 000 rpm), and the supernatant was decanted. 5 mL of water was added, the suspension was mixed, and the supernatant was removed. The washing procedure was repeated thrice. Next, the product was washed seven times with 5 mL of diethyl ether and dried at 22 °C for 24 h in a vacuum oven. Yield: 1.92 g (55%). Elemental analysis (%): theoretical: C, 38.00; H, 3.72; N, 14.77; experimental: C, 38.25; H, 3.98; N, 14.21. ^1^H NMR (400 MHz, CDCl_3_): δ (ppm) 8.16 (s, 2H), 8.07 (s, 2H), 7.77 (d, *J* = 9.0 Hz, 4H), 7.00 (d, *J* = 9.0 Hz, 4H), 4.49 (s, 6H), 4.16 (m, 4H), 4.06–3.93 (m, 4H) (see Figure S6 in the Supporting Information). FT-IR bands ν (cm^–1^), ***trans*-1**/***trans*-2** (Figure S7): 3415/3292 (O–H stretching), 3113/3124 (C–H stretching, aromatic), 2937/2940 (C–H stretching), 1596/1600, 1543/1536 (C–C aromatic), 1499/1497 (N=N stretching), 1446/1444 (C–H bending), 1246/1246 (C–O stretching), 1024/1019 (C–O – C stretching). HR-MS (ESI^+^) *m*/*z* calcd for C_24_H_28_Cl_2_N_8_O_4_Pt [M + Na]^+^, 781.51; found, 781.11.

All compounds are >95% pure by elemental and NMR analyses (since the Pt complexes are susceptible to decomposition on a chromatographic column, HPLC is not the optimal method for the determination of their purity).

### Apparatus

NMR spectra of both photoisomers of **1** and **2** were measured in CDCl_3_ or DMF-*d*_7_ using either a 400 or 600 MHz spectrometer (JEOL). FT-IR spectra of ***trans*-1** and ***trans*-2** were collected by using a Nicolet iS10 spectrophotometer (Thermo Scientific). UV–vis spectra were recorded using a Varian Cary 50 UV–vis spectrophotometer (Agilent Technologies). HPLC analyses were carried out with an HPLC Dionex UltiMate 3000 equipped with a diode array (Sunnyvale, CA, USA). The analyses were carried out using an analytical Wakopak Handy ODS column (150 × 4.6 mm) and a gradient elution. Mobile phase A was 0.1% HCOOH in water, and mobile phase B was 80% ACN in water (v/v). A linear gradient from 0 to 70% of mobile phase B in 20 min, at a flow rate of 1 mL/min, was applied. LC–MS measurements were performed using a UPLC-MS/MS system composed of ACQUITY UPLC and TQD mass spectrometers, and a TripleTOF 5600+ (SCIEX, Framingham, MA, USA) high-resolution mass spectrometer coupled to a UPLC system (Nexera X2, Shimadzu, Canby, OR, USA). The HR-MS measurement was performed using a MicroTOF II mass spectrometer (Bruker, Bremen, Germany) equipped with a time-of-flight (TOF) analyzer. The MS detection was performed using the positive ion mode (ESI^+^), and the profile spectra were acquired within the mass range of 50–3000 *m*/*z*. The ESI conditions were as follows: nebulizer pressure 0.4 bar, dry gas 4.0 L/min heated up to 180 °C, and capillary voltage −4500 V. Mass calibration was carried out using sodium formate clusters according to the procedure given by a manufacturer. Data were collected by Compass DataAnalysis 3.2 software (Bruker). Expected ions attributed to analytes were predicted by the IsotopicPattern software (Bruker, Germany). Before the analysis, samples were dissolved in acetonitrile (LC–MS grade, Honeywell). The solutions were irradiated with 365 and 530 nm LED plates (Bio Research Center, Japan). Cytotoxicity was measured using a microplate reader (Synergy HTX, BioTek, Winooski, VT, USA).

### Circular Dichroism Spectroscopy

All CD measurements were taken using a J-710 spectropolarimeter (JASCO Co., Japan) equipped with a circulating water bath (JULABO Labortechnik GmbH, Germany). Spectra were recorded at 25 °C with a data pitch of 0.5 nm and a bandwidth of 2 nm. Each spectrum was the result of three averaged scans. The spectrum of the appropriate solvent was subtracted.

### Interaction of **2** with BSA

BSA solutions were titrated with both photoisomers of the complex in PBS using an automatic pipette. A 10 mM solution of the complex in DMSO was used for titration. The initial protein concentration ranged between 40 and 200 μM. The concentration of the complex increased by 10–50 μM per each titration step. After each addition of the titrant, the CD spectrum was measured in the range of 300–450 nm with an optical path length of 0.1 or 1 cm. The scanning speed was 100 nm/min. The binding constant was determined from changes in the summed signal in the range of 350–450 nm. The analysis was performed by using the one-binding-site model assuming a 1:1 interaction stoichiometry. Global analysis of three independent titrations was performed assuming a shared value of the equilibrium constant (*K*_D_) using OriginPro software (Version 2021b, OriginLab Corporation, Northampton, MA, USA).

### Interaction of **2** and Cisplatin with DNA

To a 230 μM bp solution of calf-thymus DNA in 10 mM sodium phosphate, pH 7.4, 10 mM solutions of both photoisomers of the complex and cisplatin in DMSO were added to a concentration of 250 μM. CD spectra were measured over time until a stable signal was obtained. For the analysis of interaction kinetics, the measurement was carried out in duplicate by recording the signal at 275 nm for 1 h with a resolution of 5 s at 25 °C. The process was initiated manually by adding a portion of the compound using an automatic pipette.

### Cytotoxicity Tests

Cytotoxicity studies of the resulting photocomplex and cisplatin were performed on the normal and cancer cell lines: murine mammary normal (NMuMG, ATCC CRL-1636), murine melanoma (B16-F10, ATCC CRL-6323), murine mammary cancer (4T1, ATCC CRL-2539), and human ovary cancer (A2780, 93112519, Merck). Cells were cultured on Petri dishes in a culture medium (for A2780: RPMI-1640 + 2 mM glutamine, for the rest: DMEM (high glucose, for NMuMG extra 10 μg/mL insulin was added) supplemented with 10% v/v fetal bovine serum (FBS), at 37 °C in a humidified atmosphere containing 5% v/v CO_2_. The cells were then seeded in 96-well plates and cultured for 24 h. After this time, the cells were treated with both photoisomers of **1**, **2**, or cisplatin in 1% v/v solution of DMSO in the medium (obtained by diluting the solution in DMSO, with suitable serum content) at different concentrations and incubated for 24 or 48 h. The solution of the complex in DMSO was always diluted with PBS immediately after preparation and promptly supplemented to the cell cultures. Following removal of the media, a 100 μL solution of MTT (3-[4,5-dimethylthiazol-2-yl]-2,5-diphenyltetrazolium bromide) in the medium to each well was added and kept in the dark for 4 h at 37 °C. Then, the MTT solution was gently aspirated and 100 μL of DMSO/isopropanol 1:1 v/v was added to each well. After dissolving the crystals of purple formazan, the absorbance at 570 nm was measured using a plate reader. Measurements were made in triplicate, *p < 0.05.

### Annexin V Binding Assessment

Annexin V-Cy3 Apoptosis Detection Kit (Merck) was used following the manufacturer’s protocol to determine the mechanism of cellular death caused by both photoisomers. Briefly, the 4T1 cells were seeded on coverslips in 6-well plates and incubated with both photoisomers at the concentration of 33 μM for 1 h at 37 °C. Next, cells were washed twice with 1 mL of PBS and three times with 50 μL of binding buffer (1 mM HEPES pH 7.5, 14 mM NaCl, and 0.25 mM CaCl_2_). Then, 50 μL of the double-label staining solution (1 μg/mL of AnnCy3 and 500 μM of 6-CFDA in binding buffer) was added, and cells were incubated for 10 min at RT. After staining, cells were washed five times with 50 μL of binding buffer, and coverslips were mounted on glass slides and sealed for confocal imaging. Fluorescent images were acquired using an A1-Si Nikon (Tokyo, Japan) confocal laser scanning system coupled with a Nikon inverted microscope Ti-E and processed using NIS-Elements AR 3.2 software (Nikon Europe BV, Amsterdam, The Netherlands).

### Determination of Low-Molecular-Weight Cellular Thiols

The 4T1 cells were seeded in Petri dishes and cultured for 72 h to 90% confluency. After this time, the cells were treated with both photoisomers of 2 in 1% v/v solution of DMSO in the medium (without serum) at a concentration of 11.8 μM and incubated for 2.5 h. The control experiment was performed with cells treated with a 1% v/v solution of DMSO in the medium. For RP-HPLC analysis, pellets of cells (3.5–5 × 10^6^ cells) were suspended in 0.25 mL of 0.9% NaCl/70% perchloric acid/1 mM bathophenanthroline–disulfonic acid disodium salt and sonicated three times for 5 s at 4 °C. The sediment was separated by centrifugation at 1600 *g* at 4 °C for 10 min, and the supernatant was stored at −80 °C until analysis. The levels of low-molecular-weight thiols, i.e., reduced (GSH) and oxidized (GSSG) glutathione, and cysteine, in the incubation mixtures were determined using the RP-HPLC method of Dominic and others^60^ with modifications described by Bronowicka-Adamska and others.^61^ The samples were separated on a 4.6 mm × 250 mm Luna C18 (5 μm) column (Phenomenex, Warsaw, Poland) with a Phenomenex Security Guard column filled with the same packing material. The chromatographic system consisted of LC-10 Atvp Shimadzu Corp. (Kyoto, Japan) pumps, four channel degassers, a column oven, a Shimadzu SIL-10 Advp autosampler, and a Shimadzu Corp. SIL-10 SPD-M10Avp-diode array detector. LabSolutions LC software (Shimadzu, Kyoto, Japan) was used for system operation and data collection.

### Protein Content Determination

The pellets of cells (3.5–5 × 10^6^ cells) were suspended in 0.1 M phosphate buffer, pH 7.5, in proportion to **1** million cells/0.04 mL of the buffer and sonicated three times for 5 s at 4 °C. After centrifugation at 1600 *g* at 4 °C for 10 min, the supernatant was used for the determination of protein content. Total protein content was determined by the method of Lowry and others.^62^ The crystalline BSA was used as a standard.

### Uptake of **2** and Cisplatin by 4T1 Cells

The 4T1 cells were seeded in 6-well plates and cultured for 24 h. After this time, the cells were treated with both complex photoisomers and cisplatin as a reference drug at the concentration of 66 μM and incubated for 1–4 h in the dark at 37 °C. Cells incubated in the medium with 1% v/v DMSO in PBS served as a negative control. After that time, the medium containing the respective Pt compound was removed, the cells were washed three times with PBS, harvested by trypsinization, and centrifuged, and the supernatant was removed. The samples of cellular pellets were mineralized using concentrated nitric acid and a high-pressure mineralization technique supported by microwave radiation (Anton Paar, Ultrawave 3000). Obtained digests were diluted with deionized water up to 10 mL, and platinum was determined using an ICP–MS spectrometer (PerkinElmer, Nexion 5000). All experiments were conducted in triplicate. *p = 0.05.

### Theoretical Calculations

DFT calculations were carried out for both photoisomers of **1** ligand and **2**. All structures have been optimized with the Amsterdam density functional (ADF) program, version 2014.07.^63^ For each structure, B3LYP as an exchange–correlation functional^64,65^ was applied. The evaluation of the functional has been previously performed (see Section ST1 in the Supporting Information). The Grimme dispersion correction was used.^66^ Solvation effects were modeled with the conductor-like screening model (COSMO).^67^ The zero-order regular approximation (ZORA) to the Dirac equation was applied.^68^ For all of the atom types in the system, the triple-ζ basis set (TZP) was used. To visualize the results of geometry optimization, such as electronic features of considered systems like molecular electrostatic potential (MEP)^69^ or molecular orbitals (MOs), the ADF View package was used. Bonding between ligands and metal centers was analyzed with the Ziegler–Rauk bond energy decomposition scheme (ETS method).^70,71^ In this approach, the overall bonding energy, Δ*E*_bonding_, was calculated as Δ*E*_bonding_ = Δ*E*_orb_ + Δ*E*_steric_ + Δ*E*_disp_ = Δ*E*_orb_ + (Δ*E*_elstat_ + Δ*E*_Pauli_) + Δ*E*_disp_, where Δ*E*_bonding_ is the total interaction energy of fragments (in the geometry of the complete interacting system). The orbital interaction term, Δ*E*_orb_, describes the energetic effect related to the charge transfer between the fragments and their internal polarization (thus, leading to the formation of the optimized orbitals of the whole system), and the Δ*E*_disp_ is the dispersion correction based on the Grimme approximation. Δ*E*_steric_ can be decomposed into the electrostatic term, Δ*E*_elstat_, describing the electrostatic interaction between the unperturbed fragments and the Pauli repulsion term, Δ*E*_Pauli_ (mutual orthogonalization of the fragment orbitals, without any charge transfer between the fragments).

The CP2K software was used for MD simulations.^72^ The DFT methodology was chosen as an approach to electronic structure evaluation using the Quickstep procedure scheme^73^ with PBE as an exchange–correlation functional^74^ and the Grimme3 dispersion correction. The DZVP-MOLOPT-GTH basis set^75^ was applied for all atoms except Pt. Platinum was described by a short-range version basis set, DZVP-MOLOPT-SR-GTH.^75^ The cutoff for plane waves was setup as 350 Ry.

The simulation was carried out in a canonical ensemble (*NVT*). The time step of the simulation was 1 fs, and the temperature was set to 300 K. The full length of the simulation for both considered compounds and their photoisomers was 25,000 fs, and to obtain a relatively constant temperature, the Nose–Hoover thermostat was applied.^76^ All simulations obtained from the applied methodology were visualized and analyzed using the Visual Molecular Dynamics (VMD) software.^77^

## Supporting Information Available

Results of EA of 1 and 2; HPLC chromatogram of ***trans*-2**;^1^H NMR spectra of 1 and ***trans*-2** in CDCl_3_, UV–vis spectra of ***trans*-1** and ***cis*-1** in 1% DMSO in PBS; ^1^H NMR spectra of nonirradiated and irradiated 1 and the complex; FT-IR spectra of ***trans*-1** and ***trans*-2**; LC–MS of ***trans*-2**; HR-MS spectrum of ***trans*-2**; the mass spectra of ***cis*-1**, ***trans*-1**, ***trans*-2**, and the complex isotopic profile obtained in LC–MS analysis of the ***trans*-2** solutions in DMSO; UV–vis spectra of ***trans*-2** kept in the dark at 25 and 37 °C in 1% v/v DMSO in PBS; description of the crystallization attempts; cytotoxicity of **1** in B16–F10, 4T1, and NMuMG cells after 24 and 48 h in serum- and serum-free conditions; cytotoxicity of ***trans*-2**, ***cis*-2**, and cisplatin in B16–F10, 4T1, and NMuMG cells after 24 and 48 h in serum-free conditions; cytotoxicity of ***trans*-2** and ***cis*-2** in the A2780 ovary cancer cell line after 24 h in serum-free conditions; determination of **1**’s solubility in water; evaluation of the exchange–correlation functional of **1**; comparison of selected structural properties of **1** and azobenzene; and dynamic stability of the Pt(1)_2_ group (PDF)
Molecular formula strings (CSV)
*Ab initio* MD of the *cis* complex photoisomer (MPG)
*Ab initio* MD of the *trans* complex photoisomer (MPG)

## Supplementary Material

jm4c01575_si_002.csv

jm4c01575_si_003.mpg

jm4c01575_si_004.mpg

## Abbreviations used

4T1: cancer murine breast cells
6-CFDA: 6-carboxyfluorescein diacetate
AAP: arylazopyrazole
ADF: Amsterdam density functional
ATCC: American Type Culture Collection
ACN: acetonitrile
AMD473: *cis*-amminedichloro(2-methylpyridine) platinum(II), picoplatin
AnnCy3: Annexin V-Cy3 conjugate
ATR: attenuated total reflectance
B16-F10: murine melanoma cells
B3LYP: Becke, 3-parameter, Lee–Yang–Parr
COSMO: conductor-like screening model
CD: circular dichroism
CDDP: *cis*-diamminedichloroplatinum(II)
CRL: cell repository line
CTR1: copper transporter 1
DMEM: Dulbecco’s modified Eagle’s medium
DZVP: double-zeta valence with polarization
EA: elemental analysis
ESI: electrospray ionization
ETS: extended transition state
FBS: fetal bovine serum
FT: Fourier transform
GSH: reduced glutathione
GSSG: oxidized glutathione
GTH: Goedecker–Teter–Hutter pseudopotentials
HEPES: 4-(2-hydroxyethyl)-1-piperazineethanesulfonic acid
HPLC: high-performance liquid chromatography
HR-MS: high-resolution mass spectrometry
ICP–MS: inductively coupled plasma mass spectrometry
IR: infrared
LC–MS: liquid chromatography–mass spectroscopy
LED: light-emitting diode
MEP: molecular electrostatic potential
MOLOPT: molecularly optimized
MTT: 3-(4,5-dimethylthiazol-2-yl)-2,5-diphenyltetrazolium bromide
NMuMG: normal murine breast cells
NVT: number of particles, volume, and temperature
NMR: nuclear magnetic resonance
PBE: Perdew–Burke–Ernzerhof
PDT: photodynamic therapy
PS: photoswitch
PSS: photostationary state
RP: reverse phase
RPMI: Roswell Park Memorial Institute
RT: room temperature
SR: short-range
TOF: time of flight
TZP: triple-zeta with polarization
UPLC: ultraperformance liquid chromatography
VMD: Visual Molecular Dynamics
XRD: X-ray diffraction
ZORA: zero-order regular approximation
