# Supplementary material for: Light-Controlled Anticancer Activity and Cellular Uptake of a Photoswitchable Cisplatin Analogue
Source: J Med Chem. 2024 Oct 24;67(21):19103–20. doi: 10.1021/acs.jmedchem.4c01575 (PMC11571217; doi:10.1021/acs.jmedchem.4c01575)
Supplement: Supplementary file 1 — jm4c01575_si_001.pdf [file jm4c01575_si_001.pdf]

Supporting Information

Light-controlled anticancer activity and cellular uptake of a photoswitchable cisplatin analog

Marta Stolarek[1,2], Kamil Kamiński[1], Marta Kaczor-Kamińska[3], Magdalena Obłoza[1], Piotr Bonarek[4], Anna Czaja[5], Magdalena Datta[5], Wojciech Łach[1], Mateusz Brela[1], Artur Sikorski[5], Janusz Rak[5], Maria Nowakowska[1], Krzysztof Szczubiałka[1]*

[1] Faculty of Chemistry, Jagiellonian University, Gronostajowa 2, 30-387 Cracow, Poland

[2] Jagiellonian University, Doctoral School of Exact and Natural Sciences, Łojasiewicza 11, 30-348, Cracow, Poland

[3] Chair of Medical Biochemistry, Jagiellonian University, Collegium Medicum, Kopernika 7C, 31-034, Cracow, Poland

[4] Faculty of Biochemistry, Biophysics and Biotechnology, Jagiellonian University, Gronostajowa 7, 30-387 Cracow, Poland

[5] Faculty of Chemistry, University of Gdańsk, Wita Stwosza 63, 80-308 Gdansk, Poland

Email of the corresponding author: szczubia@chemia.uj.edu.pl

List of Figures **Page #**

Figure S1. Synthesis scheme of the complex. 5

Figure S2. HPLC chromatogram of *trans*-2. Analysis conditions: Dionex Ultimate 3000 HPLC system (Thermofisher Scientific) with DAD detector, λ=260 nm, C18 column (Wakopak Handy ODS; 150×4.6 mm; 5 µm, 110 Å). Analysis program: phase A: 0.1% HCOOH, phase B: 80% ACN, 0-70% phase B in 20 min at 25 °C, flow rate 1 ml/min. Sample volume 20 µL. 5

Figure S3. ^{1}H NMR spectrum of *trans*-1 in $CDCl_3$. 6

Figure S4. UV-Vis spectra of *trans*-1 (a) and *cis*-1 (b) solution in 1% v/v DMSO in PBS ($2.52\cdot10^{-5}$ M) irradiated with 365 and 530 nm light, respectively. 6

Figure S5. ^{1}H NMR (400 MHz, DMF-d_7) spectra of a) nonirradiated 1 (*trans*), b) 1 irradiated with 365 nm light (*cis*) and then c) with 530 nm light (*trans*), d) the nonirradiated complex (*trans*-2), e) the complex irradiated with 365 nm light (*cis*-2) and then f) with 530 nm light (*trans*-2). 8

Figure S6. ^{1}H NMR spectrum of *trans*-2 in $CDCl_3$ 8

Figure S7. Attenuated total reflectance FT-IR spectra (ATR-FT-IR) of *trans*-2 (solid line) and *trans*-1 (dotted line) normalized for the same intensity at the strongest absorption band (at 1246 cm^{-1}) to facilitate visualization of the possible shifts in the band frequency upon complexation. 8

Figure S8. LC chromatogram of the solution of the synthesis product obtained (a), the mass spectrum of the chromatogram peak at the retention time of 5.73 min (b), and the mass spectrum of the chromatogram peak at the retention time of 4.57 min (c). 9

Figure S9. The region of the high-resolution MS spectrum which corresponds (taking into account that ions are $[M+Na]^+$ adducts) to the assumed molecular mass of the complex (a) and the theoretical isotopic profile based on expected elemental composition of the complex ($C_{24}H_{28}N_8O_4Cl_2Pt$) (b).........9

Figure S10. The mass spectra of *cis*-1 (a); *trans*-1 (b); *trans*-2 (c) and the complex isotopic profile (d) obtained in LC-MS analysis of the *trans*-2 solutions in DMSO. 10

Figure S11. UV-Vis spectra of *cis*-2 kept in the dark at 25°C (a) and 37°C (b) in 1% v/v DMSO in PBS. 11

Figure S12. Microscopic photographs of the title compound before (a) and after crystallization from isopropanol (magnification x40) (b). 12

Figure S13. Cytotoxicity of 1 in B16-F10 murine melanoma cell line after 24 (upper row) and 48 h (lower row) of cell culture in serum (left column) and serum-free (right column) conditions determined with the MTT test. Culture medium was Dulbecco′s Modified Eagle′s Medium - high glucose. Statistically significant differences are indicated with asterisks above the bars (difference from the control) and above the brackets (difference between the photoisomers), *$p<0.05$..................12

Figure S14. Cytotoxicity of 1 in 4T1 murine mammary cancer cell line after 24 (upper row) and 48 h (lower row) of cell culture in serum (left column) and serum-free (right column) conditions determined with the MTT test. Culture medium was Dulbecco′s Modified Eagle′s Medium - high glucose. Statistically significant differences are indicated with asterisks above the bars (difference from the control) and above the brackets (difference between the photoisomers), *$p < 0.05$...............13

Figure S15. Cytotoxicity of 1 in NMuMG murine mammary cell line after 24 (upper row) and 48 h (lower row) of cell culture in serum (left column) and serum-free (right column) conditions determined with the MTT test. Culture medium was Dulbecco′s Modified Eagle′s Medium - high glucose. Statistically significant differences are indicated with asterisks above the bars (difference from the control) and above the brackets (difference between the photoisomers), *$p<0.05$..................13

Figure S16. Cytotoxicity of *trans*-2, *cis*-2 and cisplatin in B16-F10 murine melanoma cell line after 24 (A) and 48 h (B) of cell culture in serum-free conditions determined with the MTT test. The culture medium was Dulbecco′s Modified Eagle′s Medium - high glucose. The numbers denote the *cis/trans*, cisplatin/*cis* and cisplatin/*trans* viability ratios at respective concentration. Statistically significant differences are indicated with asterisks above the bars (difference from the control) and above the brackets (difference between the photoisomers and between cisplatin and either of the photoisomers), *$p<0.05$. 14

Figure S17. Cytotoxicity of *trans*-2, *cis*-2 and cisplatin in T41 murine mammary cancer (A and B) and normal NMuMG (C and D) cell line after 24 (A and C) and 48 h (B and D) of cell culture in serum-free conditions determined with the MTT test. Culture medium was Dulbecco′s Modified Eagle′s Medium - high glucose which in NMuMG cultures was supplemented with 1% FBS. The numbers denote the *cis/trans*, cisplatin/*cis* and cisplatin/*trans* viability ratios at respective concentration. Statistically significant differences are indicated with asterisks above the bars (difference from the control) and above the brackets (difference between the photoisomers and between cisplatin and either of the photoisomers), *$p<0.05$. 15

Figure S18. Cytotoxicity of *trans*-2 and *cis*-2 in A2780 ovary cancer cell line after 24 h of cell culture in serum-free conditions determined based on MTT test. Test. The culture medium was RPMI-1640 + 2mM glutamine. The numbers above the bars denote the ratio of the viability for respective *cis* and *trans* photoisomers at each concentration. Statistically significant differences are indicated with

asterisks above the bars (difference from the control) and above the brackets (difference between the photoisomers), *p<0.05. 16

Figure S19. Fluorescence microscopic images of 4T1 cells subjected to the apoptosis detection test. Negative control cells a) and cells incubated with the *cis* (b) and *trans* (c) photoisomer of the complex. The green fluorescence is emitted by 6-carboxyfluorescein (6-CF) from live cells only, while only necrotic cells are labeled with annexin V - Cy3 (AnnCy3) conjugate emitting red fluorescence....19

Figure S20. UV-Vis spectra of *trans*-1 saturated solution in water diluted 30 times (solid black line) and the standard 0.015 mg/mL solution of *trans*-1 in water....19

Figure S21. Visualization of the molecular orbitals involved in the transition predicted by TD-DFT(B3LYP) calculations a) the orbital 65 (HOMO) b) the orbital 66 (LUMO). 20

Figure S22. Alignment of ground state geometries obtained from the DFT calculations performed with B3LYP and BHandHLYP exchange-correlation functionals. 22

Figure S23. Visualization of the θCNNC dihedral angle in the 1 structure. 23

Figure S24. The comparison of the internal energy changes during the 20000 steps of the performed MD simulation. 25

Figure S25. Conformations with the highest and lowest energies obtained from the *trans*-2 conformational space screening....26

Figure S26. Conformations with the highest and lowest energies obtained from the *cis*-2 conformational space screening....26

Figure S27. The plot presenting the analyzed hypothetical dihedral angle θNNNN between the four nitrogen atoms of the pyrazole heteroaromatic rings for both *trans*- and *cis* complex photoisomers....27

Figure S28. Considered hydrogen bonding between the hydroxyl group at the end of the 1 moiety and the ether oxygen in the $Pt(1)_2$ complex....27

Figure S29. Comparison of the average values of the dihedral angle corresponding to the diazo bond configuration for both isomers of the $Pt(1)_2$ systems. 28

Figure S30. The dependence of the values of the θClPtNN dihedral angle on time indicating stability of the coordination center planarity in the $Pt(1)_2$ system of both photoisomers. The atoms forming θClPtNN are shown on the right panel. Cisplatin structure was used for simplification. 29

List of Tables **Page #**

Table S1. Elemental analysis of *trans*-1 (%, averages of 2 measurements are given)....5

Table S2. Elemental analysis of *trans*-2 (averages of 3 measurements are given) 5

Table S3. Estimated LC_{50} values (M) of *trans*-2, *cis*-2 and cisplatin in various cell lines, culture times and conditions....16

Table S4. The ratios of LC_{50} values of both photoisomers and cisplatin in various cell lines, culture times and conditions. 17

Table S5. The excitation energies, oscillatory strengths and corresponding wavelengths obtained from TD-DFT calculations with B3LYP as an exchange-correlation functional....20

Table S6. The results obtained from the exchange-correlation functional validation of the TD-DFT vertical transitions calculations performed on 1....21

Table S7. The comparison of the excitation energies obtained from the calculations with different hybrid functionals. 21

Table S8. The comparison of a selected structural properties, obtained from DFT/B3LYP geometry optimization calculations for trans-cis isomers of azobenzene and *cis*/*trans* photoisomers of 1. 23

Table S9. The comparison of the obtained values of average dihedral angles describing the dynamic properties of the Pt(1)$_2$ systems in both photoisomers. 29

Table S1. Elemental analysis of *trans*-1 (%, averages of 2 measurements are given)

Theor:	C: 58.53;	H: 5.73;	N: 22.75
Exp:	C: 58.60;	H: 5.76;	N: 22.33

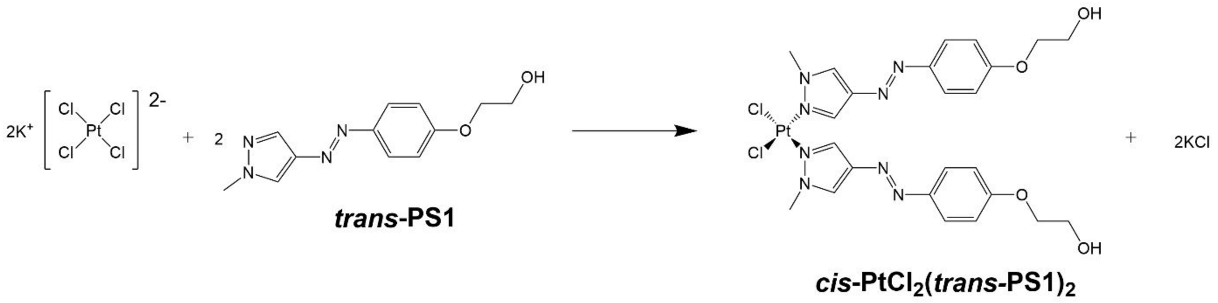

Figure S1. Synthesis scheme of the complex.

Table S2. Elemental analysis of *trans*-2 (averages of 3 measurements are given)

Theor:	C: 38.00;	H: 3.72;	N: 14.77
Exp:	C: 38.25;	H: 3.98;	N: 14.21

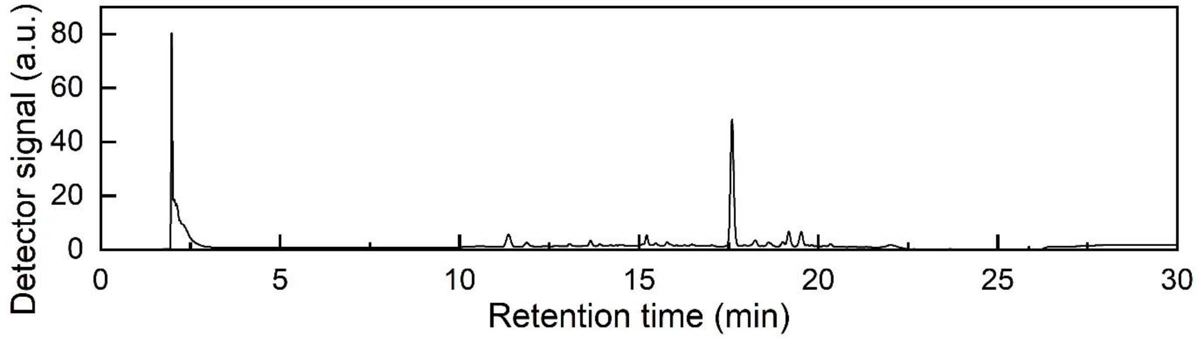

Figure S2. HPLC chromatogram of *trans*-2. Analysis conditions: Dionex Ultimate 3000 HPLC system (Thermofisher Scientific) with DAD detector, λ=260 nm, C18 column (Wakopak Handy ODS; 150×4.6 mm; 5 μm, 110 Å). Analysis program: phase A: 0.1% HCOOH, phase B: 80% ACN, 0-70% phase B in 20 min at 25 °C, flow rate 1 ml/min. Sample volume 20 μL.

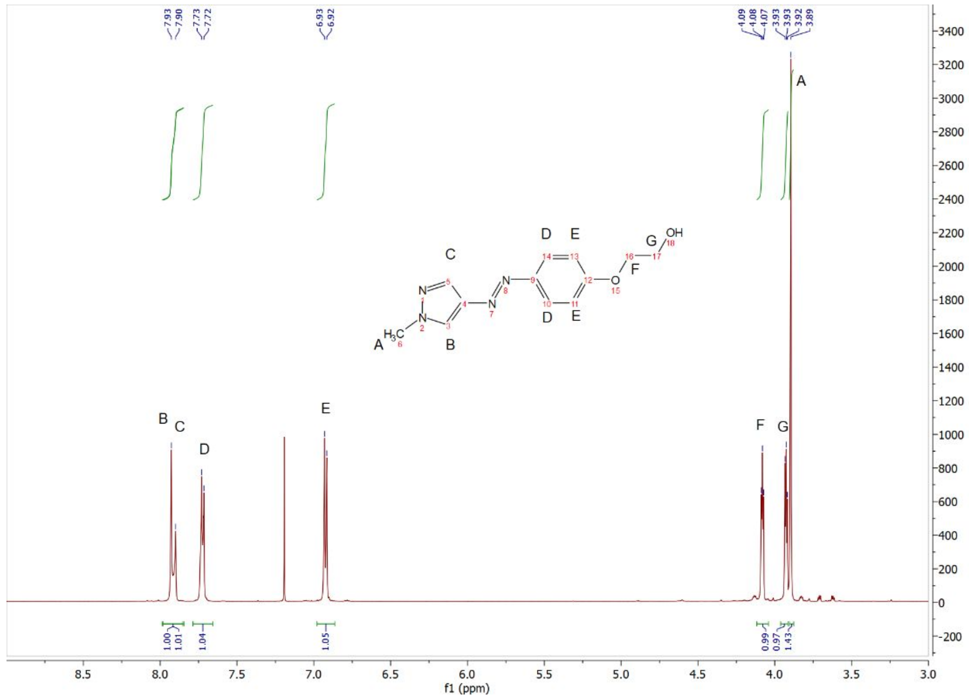

Figure S3. ^{1}H NMR spectrum of *trans*-1 in $CDCl_3$.

^{1}H NMR (600 MHz, $CDCl_3$) δ (ppm) 7.93 (s, 1H), 7.90 (s, 1H), 7.72 (d, J = 8.9 Hz, 2H), 6.92 (d, J = 8.9 Hz, 2H), 4.10 – 4.06 (m, 3H), 3.95 – 3.91 (m, 2H), 3.89 (s, 3H).

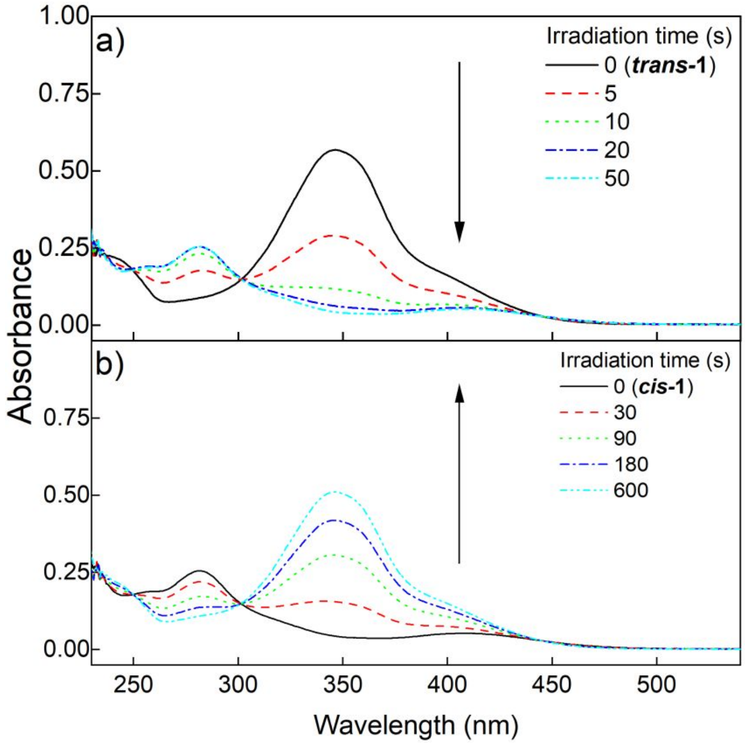

Figure S4. UV-Vis spectra of *trans*-1 (a) and *cis*-1 (b) solution in 1% v/v DMSO in PBS ($2.52 \cdot 10^{-5}$ M) irradiated with 365 and 530 nm light, respectively.

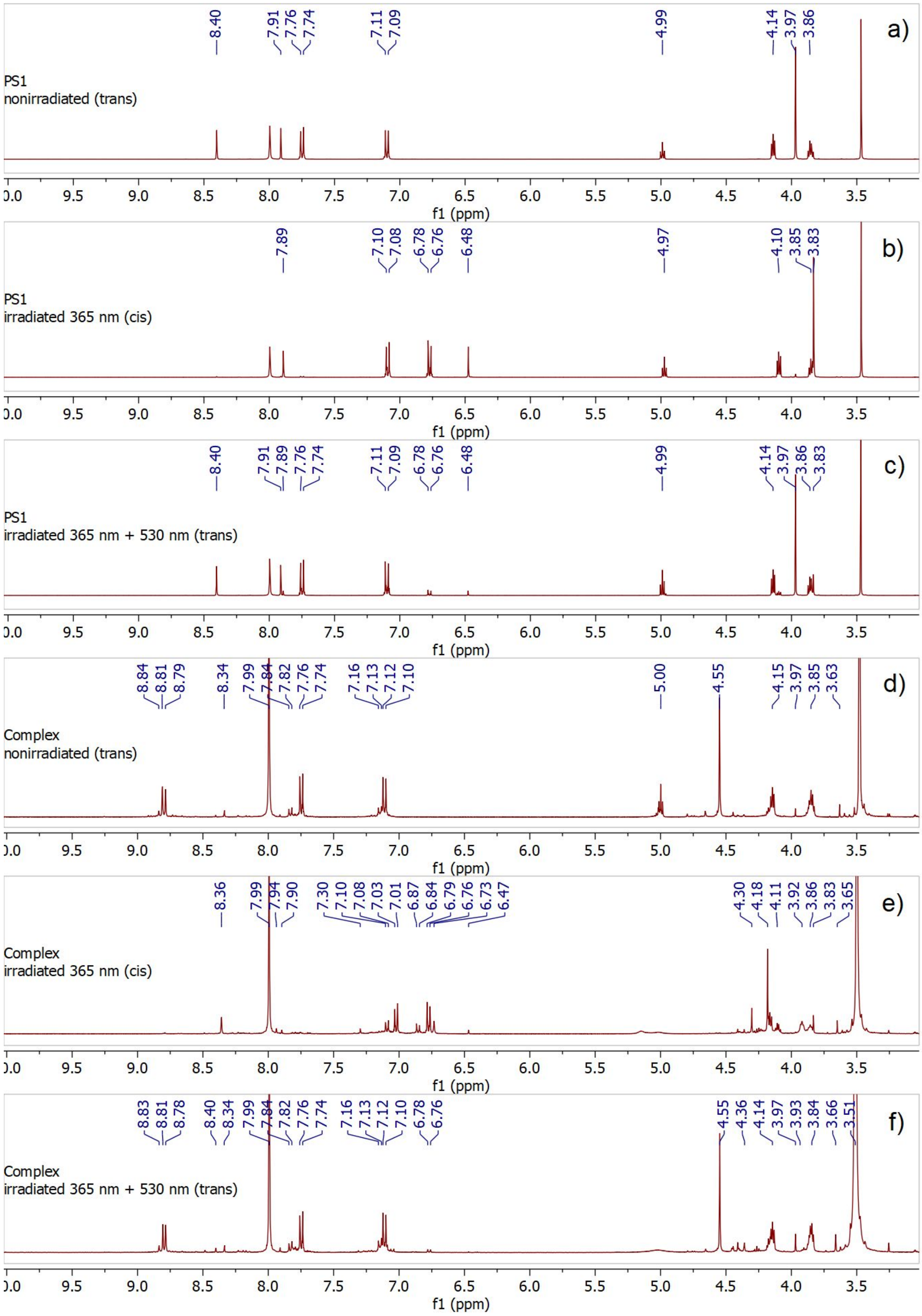
a)
PS1
nonirradiated (trans)
b)
PS1
irradiated 365 nm (cis)
c)
PS1
irradiated 365 nm + 530 nm (trans)
d)
Complex
nonirradiated (trans)
e)
Complex
irradiated 365 nm (cis)
f)
Complex
irradiated 365 nm + 530 nm (trans)
f1 (ppm)

Figure S5. ^{1}H NMR (400 MHz, DMF-d_7) spectra of a) nonirradiated 1 (*trans*), b) 1 irradiated with 365 nm light (*cis*) and then c) with 530 nm light (*trans*), d) the nonirradiated complex (*trans*-2), e) the complex irradiated with 365 nm light (*cis*-2) and then f) with 530 nm light (*trans*-2).

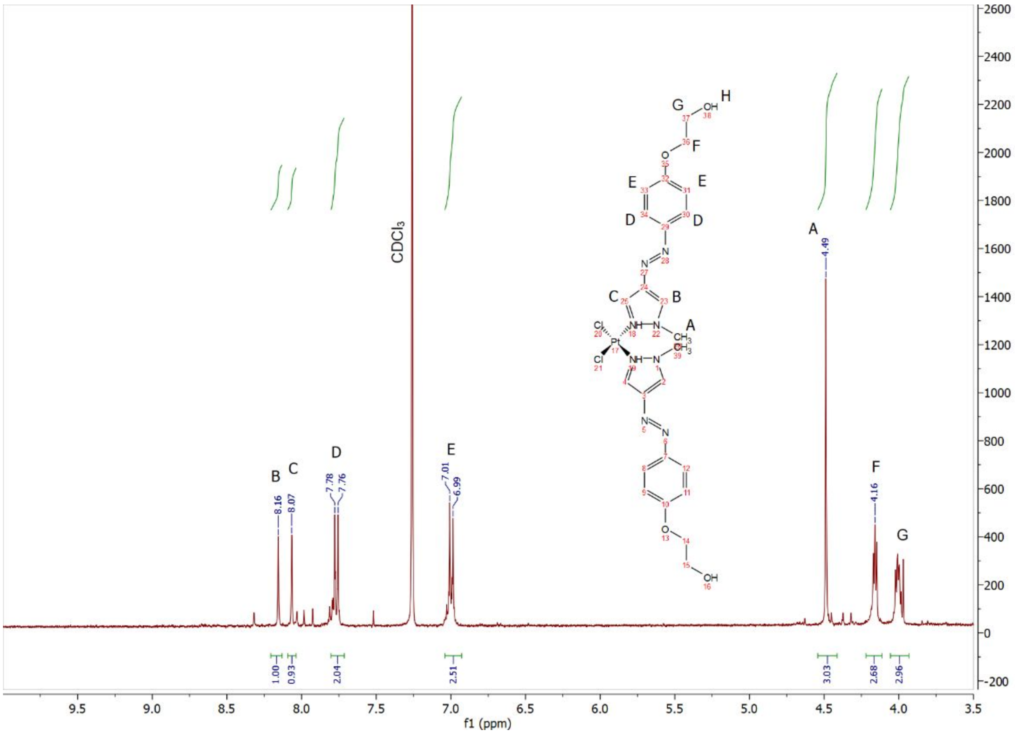

Figure S6. ^{1}H NMR spectrum of *trans*-2 in $CDCl_3$

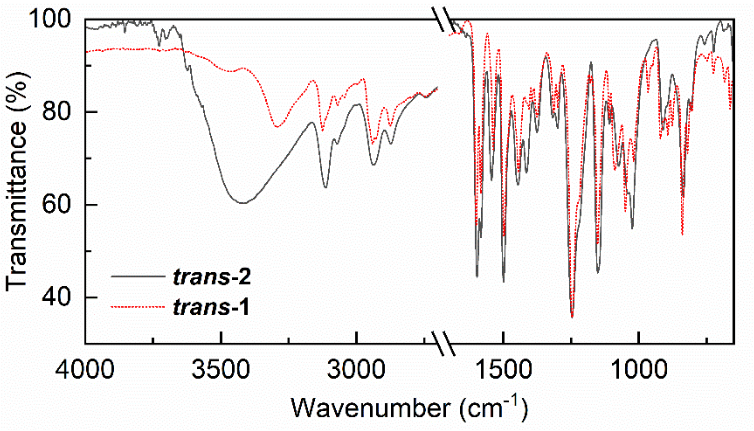

Figure S7. Attenuated total reflectance FT-IR spectra (ATR-FT-IR) of *trans*-2 (solid line) and *trans*-1 (dotted line) normalized for the same intensity at the strongest absorption band (at 1246 cm^{-1}) to facilitate visualization of the possible shifts in the band frequency upon complexation.

a)

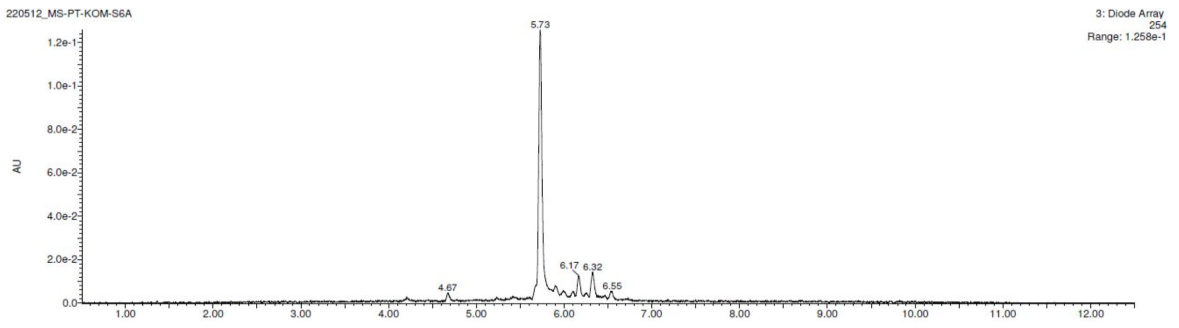

b)

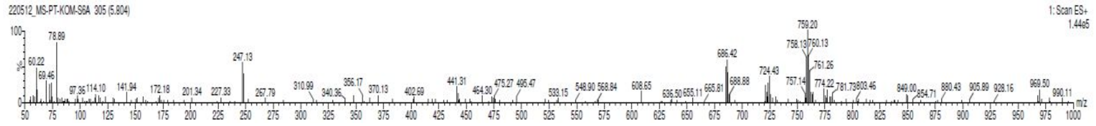

c)

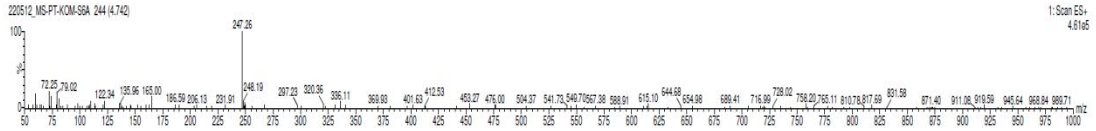

Figure S8. LC chromatogram of the solution of the synthesis product obtained (a), the mass spectrum of the chromatogram peak at the retention time of 5.73 min (b), and the mass spectrum of the chromatogram peak at the retention time of 4.57 min (c).

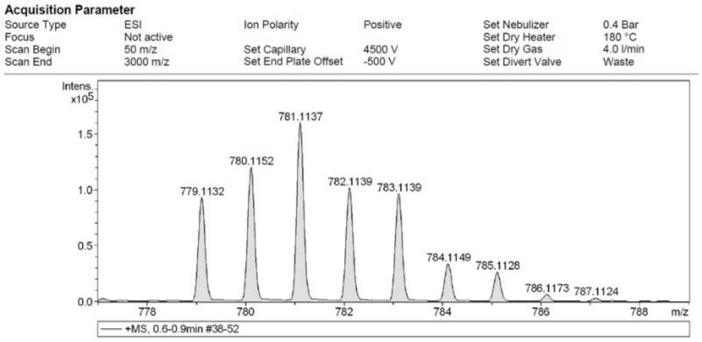

a)

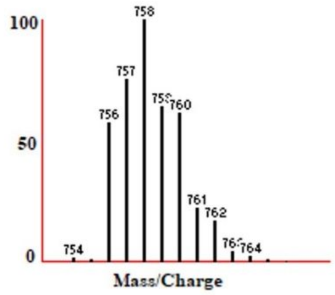

b)

Figure S9. The region of the high-resolution MS spectrum which corresponds (taking into account that ions are $[M+Na]^+$ adducts) to the assumed molecular mass of the complex (a) and the

theoretical isotopic profile based on expected elemental composition of the complex ($C_{24}H_{28}N_8O_4Cl_2Pt$) (b).

LC-MS analysis of *trans*-2 complex stability

To determine the complex stability, LC-MS analysis was performed after conditioning a solution of the complex in DMSO for several chosen periods of time up to 24 hours. MS spectrum was recorded using UPLC system (Nexera X2, Shimadzu, Canby, OR, USA) coupled with a high-resolution mass spectrometer (TripleTOF 5600+ (SCIEX, Framingham, MA, USA)). Parameters of analysis: ion source DuoSpray (ESI); positive ionization mode; spray gas pressure (N_2) 25 psi; voltage 5.5 kV; source temperature 300°C. A C18 column (Kinetex Phenomenex; C18; 2.1×150 mm; 2.6 µm, 100 Å) was used for chromatographic separation and the analysis program was set to: linear gradient 0-70% phase B for 20 minutes at 25°C, and at a flow rate of 0.3 mL/min; phase A: 0.1% HCOOH, phase B: 80% ACN. During analysis, the eluent was directed to waste for 3.5 min after injection.

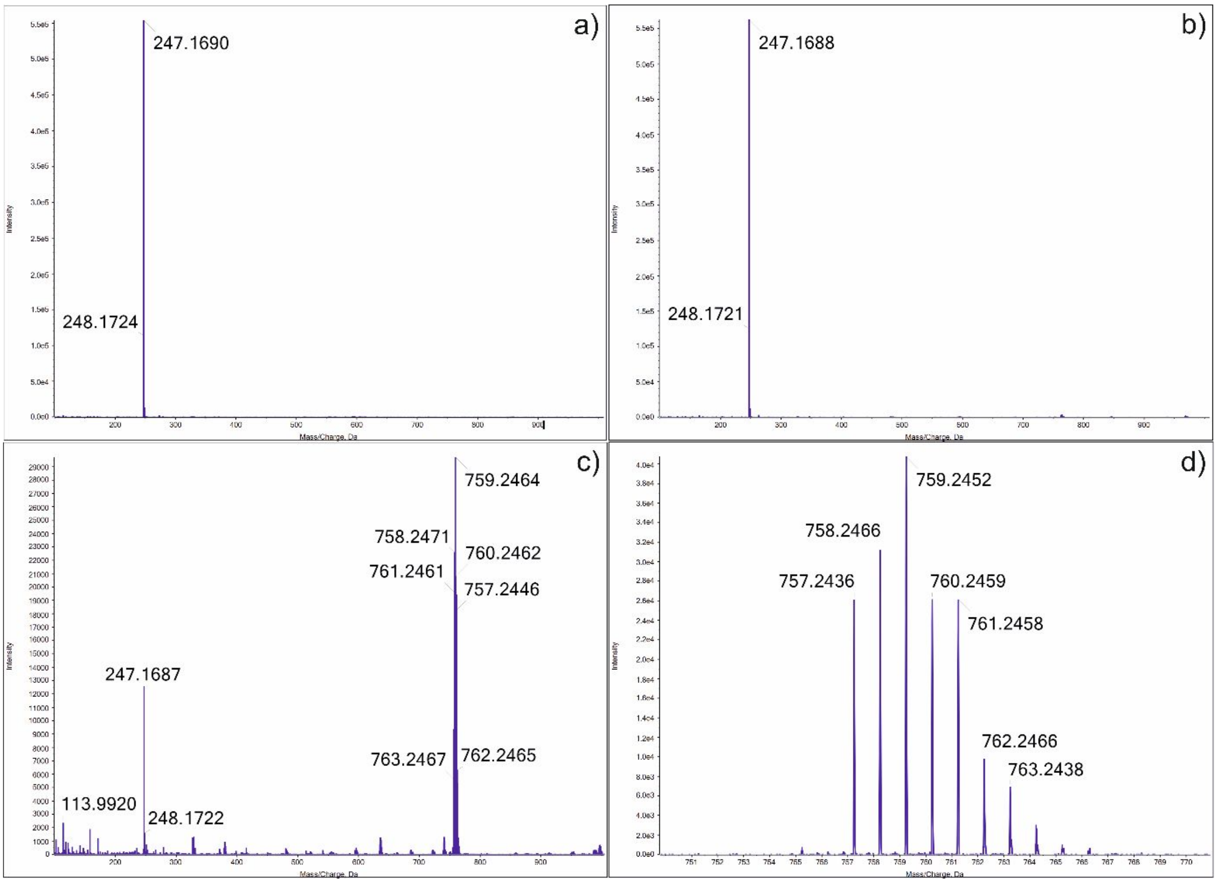

Figure S10. The mass spectra of *cis*-1 (a); *trans*-1 (b); *trans*-2 (c) and the complex isotopic profile (d) obtained in LC-MS analysis of the *trans*-2 solutions in DMSO.

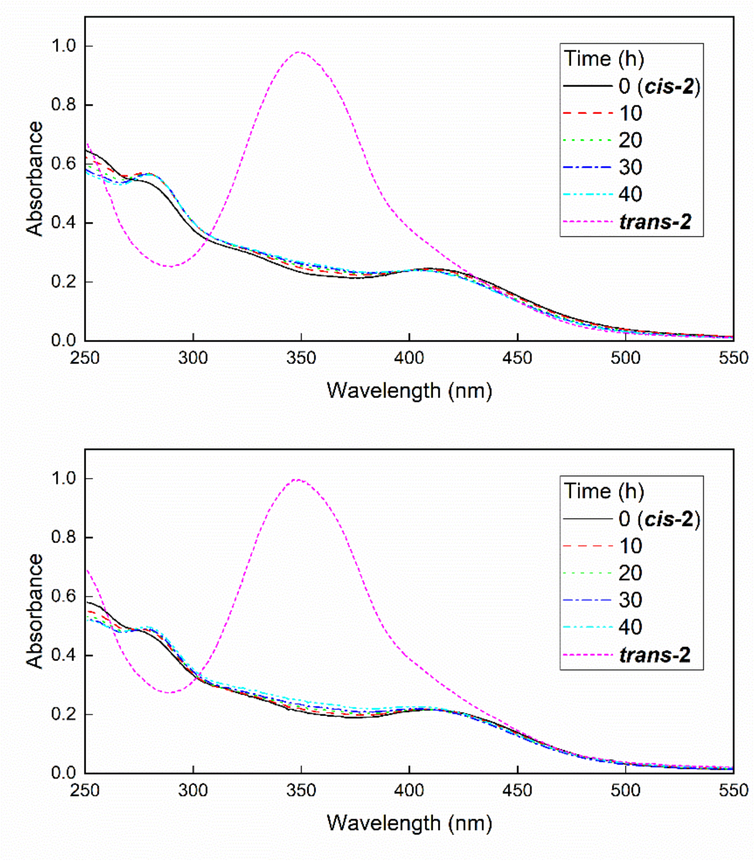

Figure S11. UV-Vis spectra of *cis*-2 kept in the dark at 25°C (a) and 37°C (b) in 1% v/v DMSO in PBS.

Crystallization attempts

Crystallization of the ($C_{24}H_{28}N_8O_4Cl_2Pt$) complex was carried out via a slow evaporation technique using the following solvents/solvent systems: water, methanol, ethanol, isopropanol, acetonitrile, acetone, dimethyl ether, 1,4-dioxane, DMF, n-hexane, cyclohexane, and mixtures of the above in various volume ratios. In all cases, an amorphous red solid was obtained (**Figure S122**), which does not diffract X-rays (preliminary experiments carried out using Oxford Diffraction Gemini R ULTRA Ruby CCD diffractometer with CuK_α radiation 1.54184 Å at temperature 291(2) K; exposure time: 100 s per frame).

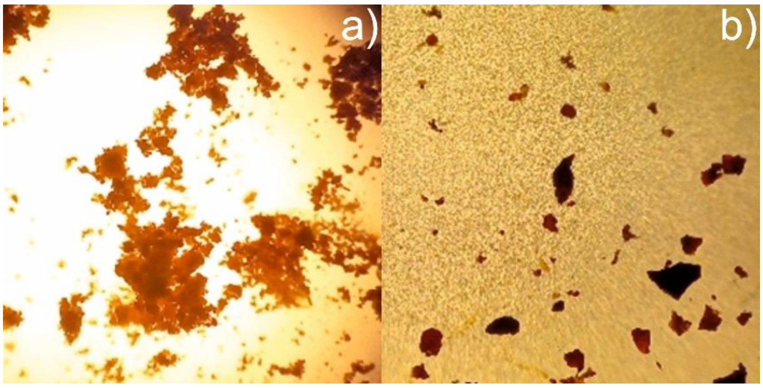

Figure S12. Microscopic photographs of the title compound before (a) and after crystallization from isopropanol (magnification x40) (b).

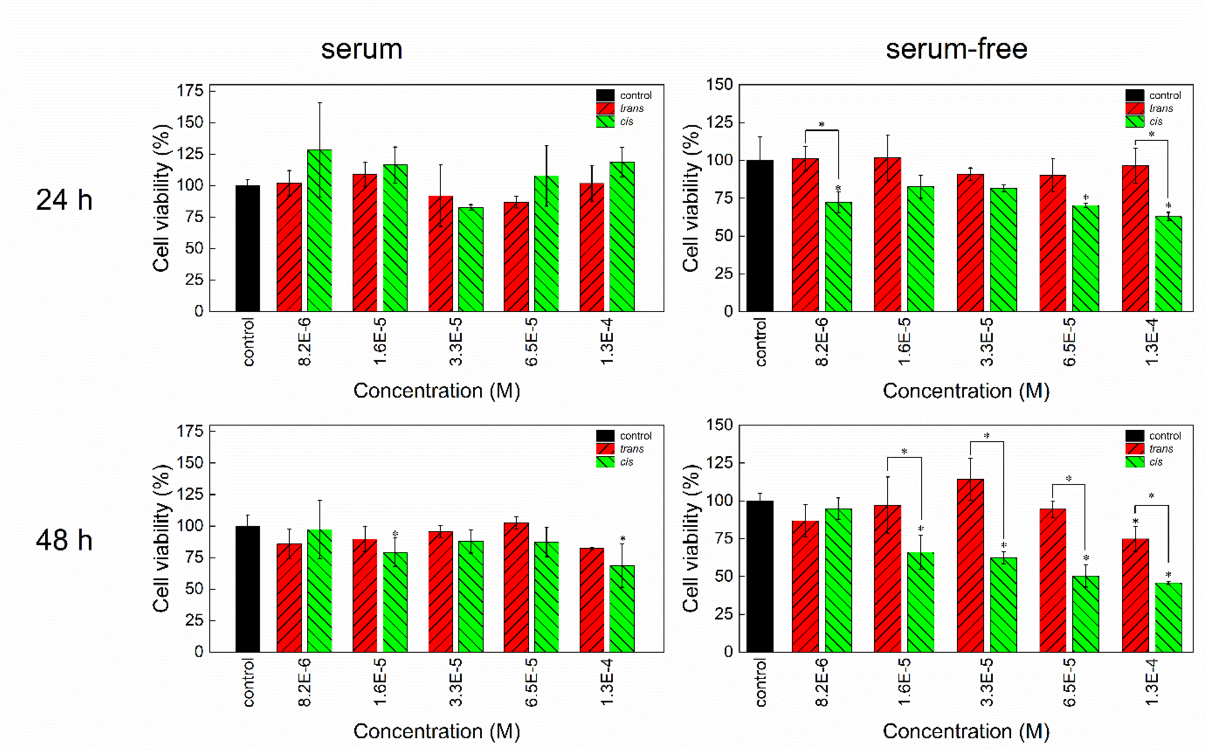

Figure S13. Cytotoxicity of 1 in B16-F10 murine melanoma cell line after 24 (upper row) and 48 h (lower row) of cell culture in serum (left column) and serum-free (right column) conditions determined with the MTT test. Culture medium was Dulbecco′s Modified Eagle′s Medium - high glucose. Statistically significant differences are indicated with asterisks above the bars (difference from the control) and above the brackets (difference between the photoisomers), *p<0.05.

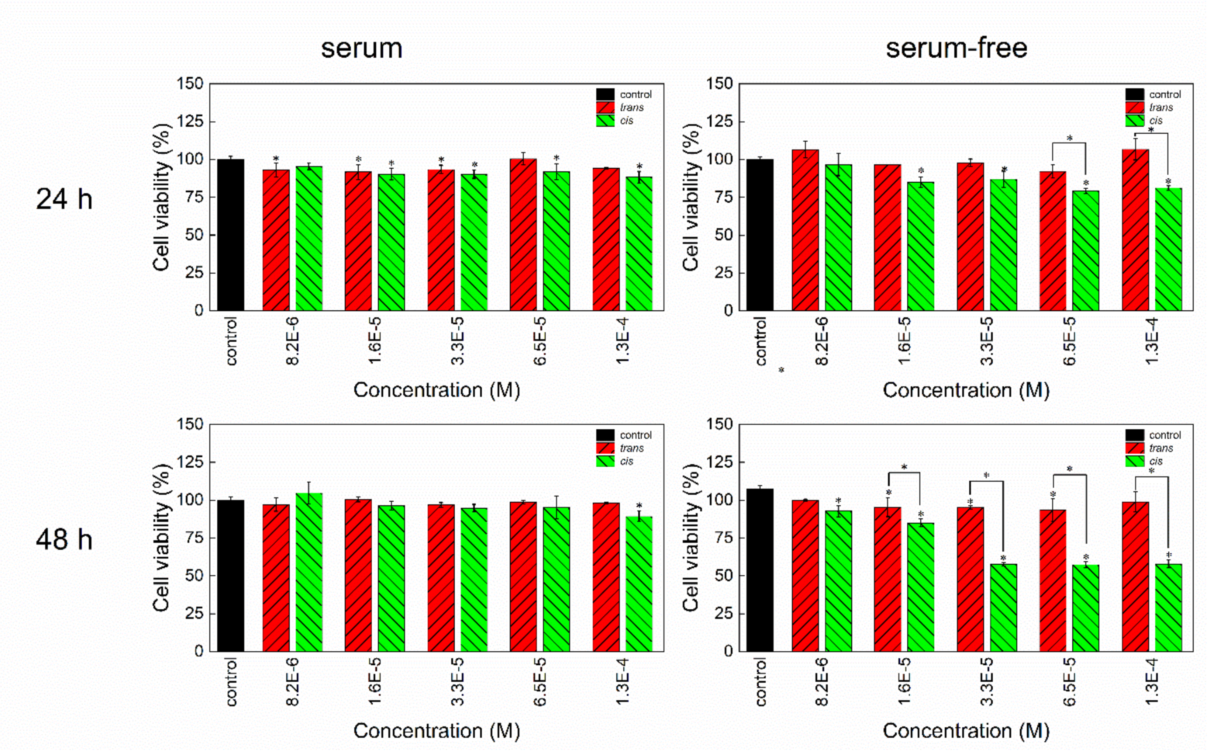

Figure S14. Cytotoxicity of 1 in 4T1 murine mammary cancer cell line after 24 (upper row) and 48 h (lower row) of cell culture in serum (left column) and serum-free (right column) conditions determined with the MTT test. Culture medium was Dulbecco′s Modified Eagle′s Medium - high glucose. Statistically significant differences are indicated with asterisks above the bars (difference from the control) and above the brackets (difference between the photoisomers), *p < 0.05.

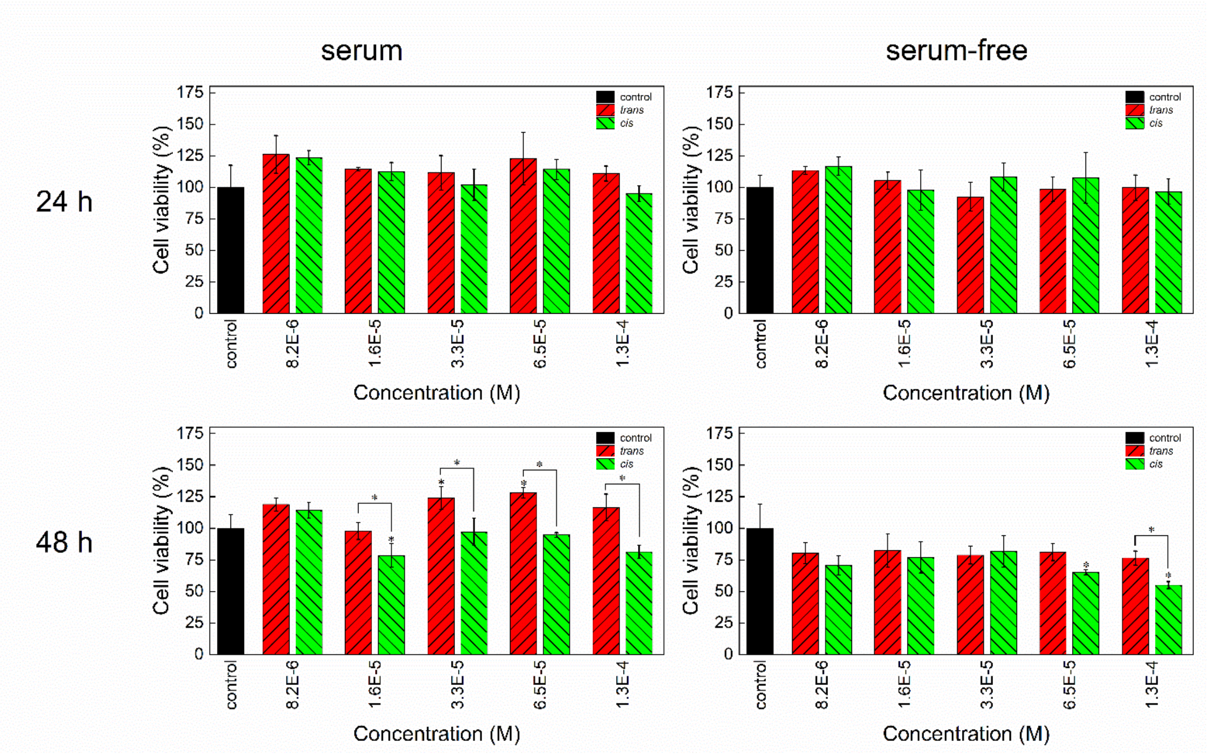

Figure S15. Cytotoxicity of 1 in NMuMG murine mammary cell line after 24 (upper row) and 48 h (lower row) of cell culture in serum (left column) and serum-free (right column) conditions determined with the MTT test. Culture medium was Dulbecco′s Modified Eagle′s Medium - high

glucose. Statistically significant differences are indicated with asterisks above the bars (difference from the control) and above the brackets (difference between the photoisomers), *p<0.05.

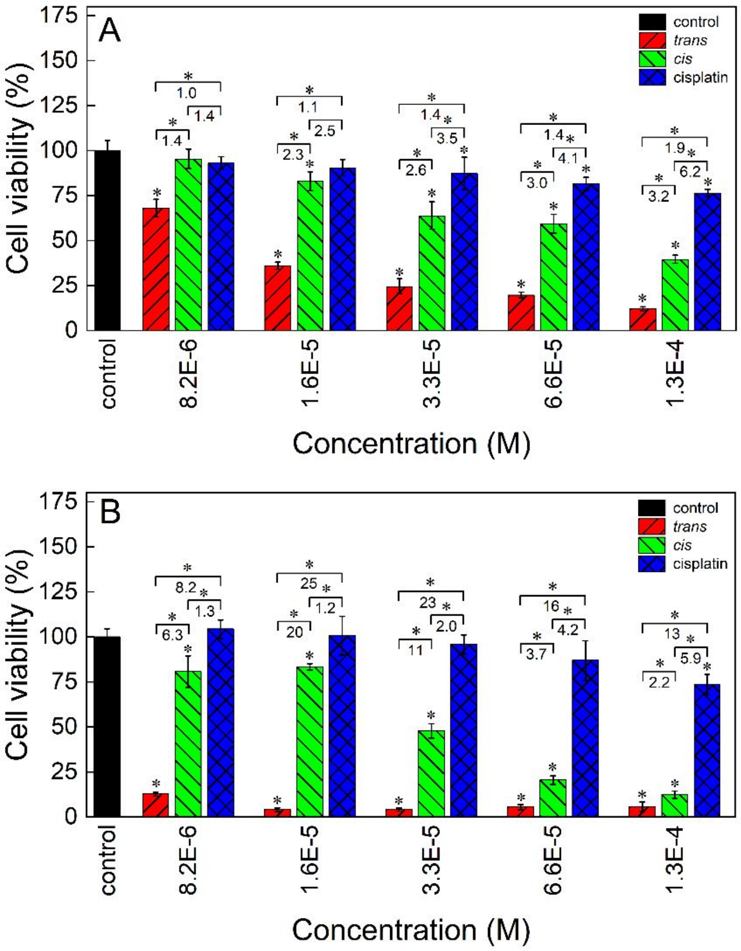

Figure S16. Cytotoxicity of *trans*-2, *cis*-2 and cisplatin in B16-F10 murine melanoma cell line after 24 (A) and 48 h (B) of cell culture in serum-free conditions determined with the MTT test. The culture medium was Dulbecco′s Modified Eagle′s Medium - high glucose. The numbers denote the *cis/trans*, cisplatin/*cis* and cisplatin/*trans* viability ratios at respective concentration. Statistically significant differences are indicated with asterisks above the bars (difference from the control) and above the brackets (difference between the photoisomers and between cisplatin and either of the photoisomers), *p<0.05.

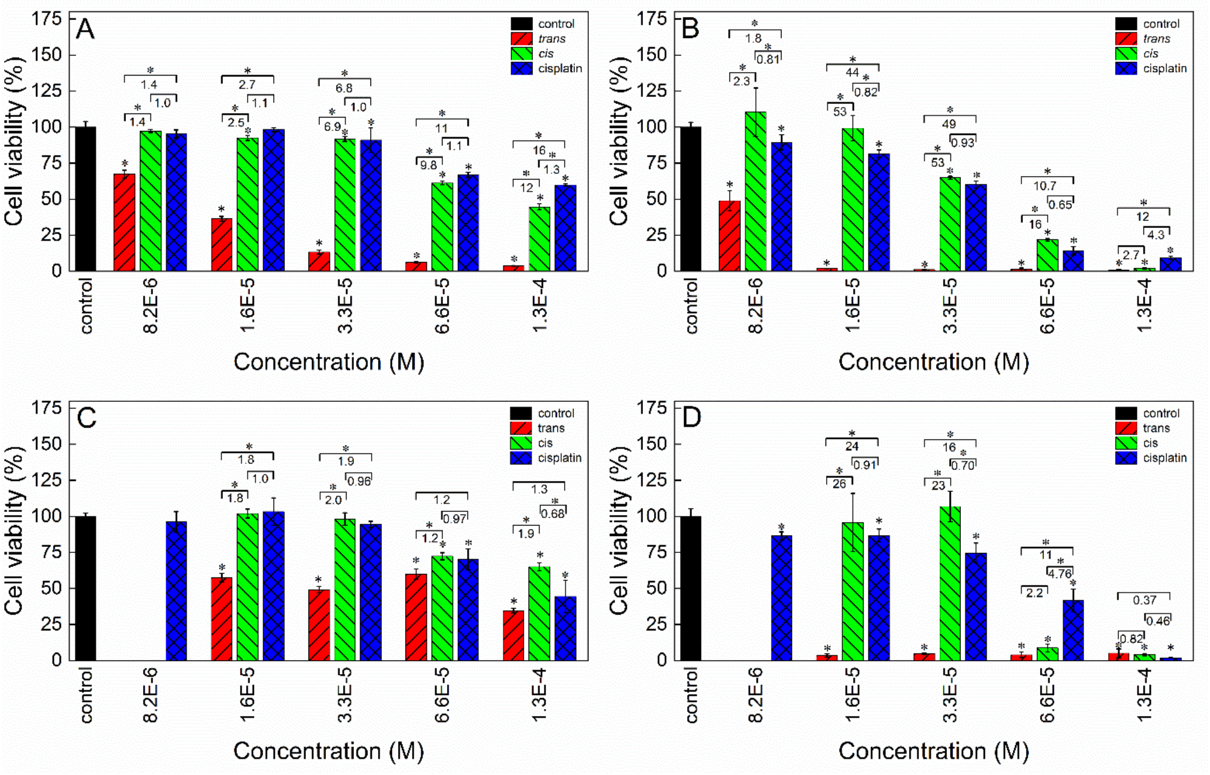

Figure S17. Cytotoxicity of *trans*-2, *cis*-2 and cisplatin in T41 murine mammary cancer (A and B) and normal NMuMG (C and D) cell line after 24 (A and C) and 48 h (B and D) of cell culture in serum-free conditions determined with the MTT test. Culture medium was Dulbecco′s Modified Eagle′s Medium - high glucose which in NMuMG cultures was supplemented with 1% FBS. The numbers denote the *cis/trans*, cisplatin/*cis* and cisplatin/*trans* viability ratios at respective concentration. Statistically significant differences are indicated with asterisks above the bars (difference from the control) and above the brackets (difference between the photoisomers and between cisplatin and either of the photoisomers), *p<0.05.

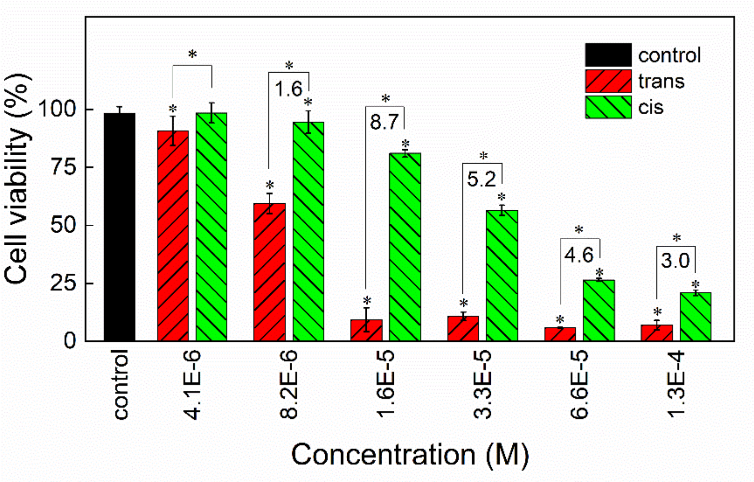

Figure S18. Cytotoxicity of *trans*-2 and *cis*-2 in A2780 ovary cancer cell line after 24 h of cell culture in serum-free conditions determined based on MTT test. Test. The culture medium was RPMI-1640 + 2mM glutamine. The numbers above the bars denote the ratio of the viability for respective *cis* and *trans* photoisomers at each concentration. Statistically significant differences are indicated with asterisks above the bars (difference from the control) and above the brackets (difference between the photoisomers), *$p<0.05$.

Comparison of the LC_{50} toxicity of the complex photoisomers and cisplatin

A complementary method to compare the cytotoxicities of the three platinum species tested, except for comparing the cell viabilities at their specific concentrations, is to calculate and compare their LC_{50} values for different cell lines, culture times and culture conditions. However, in most of the cases the full sigmoidal curve was not obtained in the concentration range tested therefore the LC_{50} values were estimated based on two-point interpolation or extrapolation and in a few cases only the minimum or maximum LC_{50} values could be given (**Table S3**). For easier visual comparison the data are shown as a heatmap with the intensity of the red color growing with increasing LC_{50} values.

Table S3. Estimated LC_{50} values (M) of *trans*-2, *cis*-2 and cisplatin in various cell lines, culture times and conditions.

Cell line	LC_{50} (M)	Culture conditions/culture time (h)			
		serum		serum-free	
		24	48	24	48

**B16-F10**	**LC_{50}(*trans*-2)**	$2.97 \cdot 10^{-5}$	$8.93 \cdot 10^{-6}$	$1.29 \cdot 10^{-5}$	$<8.16 \cdot 10^{-6}$
**murine**	**LC_{50}(*cis*-2)**	$>1.32 \cdot 10^{-4}$	$3.25 \cdot 10^{-5}$	$9.75 \cdot 10^{-5}$	$3.20 \cdot 10^{-5}$
**cancer**	**LC_{50}(cisplatin)**	$>1.32 \cdot 10^{-4}$	$8.49 \cdot 10^{-5}$	$>1.32 \cdot 10^{-4}$	$>1.32 \cdot 10^{-4}$
**4T1**	**LC_{50}(*trans*-2)**	$1.05 \cdot 10^{-5}$	$1.25 \cdot 10^{-5}$	$1.28 \cdot 10^{-5}$	$7.99 \cdot 10^{-6}$
**murine**	**LC_{50}(*cis*-2)**	$8.35 \cdot 10^{-5}$	$5.51 \cdot 10^{-5}$	$1.40 \cdot 10^{-4}$	$4.44 \cdot 10^{-5}$
**cancer**	**LC_{50}(cisplatin)**	$7.07 \cdot 10^{-5}$	$2.22 \cdot 10^{-5}$	$>1.32 \cdot 10^{-4}$	$4.04 \cdot 10^{-5}$
**NMuMG**	**LC_{50}(*trans*-2)**	$1.53 \cdot 10^{-5}$	$<1.63 \cdot 10^{-6}$	$9.19 \cdot 10^{-5}$	$<8.16 \cdot 10^{-6}$
**murine**	**LC_{50}(*cis*-2)**	$1.14 \cdot 10^{-4}$	$6.03 \cdot 10^{-5}$	$>1.32 \cdot 10^{-4}$	$5.21 \cdot 10^{-5}$
**normal**	**LC_{50}(cisplatin)**	$3.97 \cdot 10^{-5}$	$2.20 \cdot 10^{-5}$	$1.18 \cdot 10^{-4}$	$5.76 \cdot 10^{-5}$
**A2780 human**	**LC_{50}(*trans*-2)**			$9.74 \cdot 10^{-6}$	
**cancer**	**LC_{50}(*cis*-2)**			$4.01 \cdot 10^{-5}$	

As expected, the LC_{50} values of all three species decreased with time[1]. Advantageously, the LC_{50} values of the *trans* photoisomer were found to be always lower compared to those of the *cis* one and always lower than those of cisplatin. On the other hand, the LC_{50} values of the *cis* photoisomer were both lower (in B16-F10) and, advantageously, higher (in 4T1 and NMuMG) than the respective values of cisplatin. The differences in the LC_{50} values among the compounds can be quantitatively expressed by the $\frac{LC_{50}(cis)}{LC_{50}(trans)}$, $\frac{LC_{50}(\text{cisplatin})}{LC_{50}(trans)}$ and $\frac{LC_{50}(\text{cisplatin})}{LC_{50}(trans)}$ ratios which are shown as a heatmap in **Table S4**. For optimal photocontrol of the complex toxicity, the value of the $\frac{LC_{50}(cis)}{LC_{50}(trans)}$ ratio should be much higher than 1. On the other hand, for the complex to be superior to the reference drug cisplatin, its *trans* and *cis* photoisomers should be respectively much more and much less toxic than cisplatin. This means that the $\frac{LC_{50}(\text{cisplatin})}{LC_{50}(trans)}$ ratio should be as higher than 1 as possible, while the $\frac{LC_{50}(\text{cisplatin})}{LC_{50}(cis)}$ ratio should be as lower than 1 as possible. However, since the ratios are interrelated: ·

$$\frac{LC_{50}(\text{cisplatin})}{LC_{50}(cis)} \times \frac{LC_{50}(cis)}{LC_{50}(trans)} = \frac{LC_{50}(\text{cisplatin})}{LC_{50}(trans)}$$

all these requirements cannot be simultaneously fulfilled.

Table S4. The ratios of LC_{50} values of both photoisomers and cisplatin in various cell lines, culture times and conditions.

Cell line	LC_{50} ratio	Culture conditions/culture time			
		serum		serum-free	
		24	48	24	48
**B16-F10**	$\frac{LC_{50}(cis)}{LC_{50}(trans)}$	>4.4	3.6	7.6	>3.9

	$\frac{LC_{50}(\text{cisplatin})}{LC_{50}(\textit{trans})}$	>4.4	9.5	≫10.2	≫16.2
	$\frac{LC_{50}(\text{cisplatin})}{LC_{50}(\textit{cis})}$	-	2.6	>1.4	>4.1
**4T1**	$\frac{LC_{50}(\textit{cis})}{LC_{50}(\textit{trans})}$	8.0	4.4	10.9	5.6
	$\frac{LC_{50}(\text{cisplatin})}{LC_{50}(\textit{trans})}$	6.8	1.8	>10.3	5.1
	$\frac{LC_{50}(\text{cisplatin})}{LC_{50}(\textit{cis})}$	0.8	0.4	>0.9	0.9
**NMuMG**	$\frac{LC_{50}(\textit{cis})}{LC_{50}(\textit{trans})}$	7.5	≫7.4	1.4	≫6.4
	$\frac{LC_{50}(\text{cisplatin})}{LC_{50}(\textit{trans})}$	2.6	≫2.7	1.3	7.1
	$\frac{LC_{50}(\text{cisplatin})}{LC_{50}(\textit{cis})}$	0.3	0.4	<0.9	1.1
**A2780**	$\frac{LC_{50}(\textit{cis})}{LC_{50}(\textit{trans})}$	4.1			

The values of the $\frac{LC_{50}(\textit{cis})}{LC_{50}(\textit{trans})}$ ratio are always significantly greater than 1 (except for serum-free 24 h NMuMG culture). They are higher than about 4 reaching almost 11 for 4T1. Moreover, they are generally greater in serum-free conditions (except serum-free 24 h NMuMG culture). The greatest difference in the LC_{50} values of cisplatin and *trans* photoisomer was found for B16-F10 cells with the corresponding LC_{50} ratios reaching more than 16. On the other hand, the most favorable $\frac{LC_{50}(\text{cisplatin})}{LC_{50}(\textit{cis})}$ ratios (i.e., indicating desired lower toxicity of the *cis* photoisomer than that of cisplatin) were found in 4T1 and in NMuMG cells for which they did not exceed 1, except for NMuMG after 48 h serum-free culture. In B16-F10 the *cis* photoisomer was much more toxic than cisplatin with the respective ratio higher than 4.

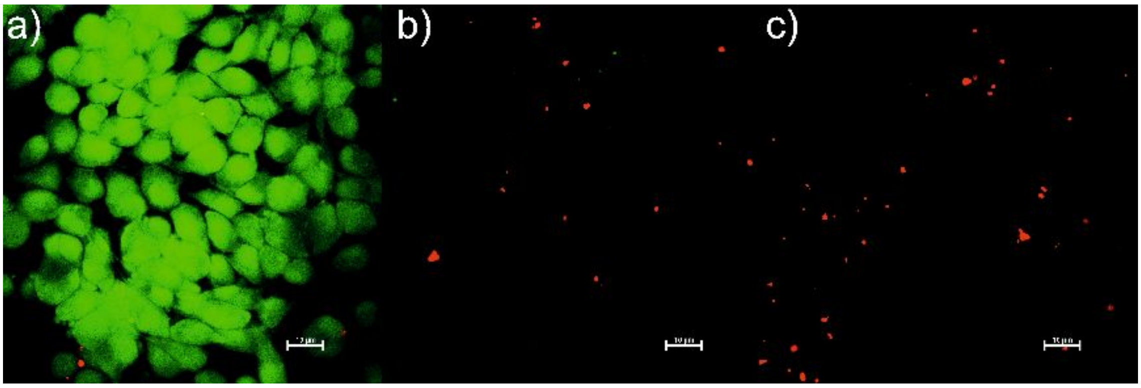

Figure S19. Fluorescence microscopic images of 4T1 cells subjected to the apoptosis detection test. Negative control cells a) and cells incubated with the *cis* (b) and *trans* (c) photoisomer of the complex. The green fluorescence is emitted by 6-carboxyfluorescein (6-CF) from live cells only, while only necrotic cells are labeled with annexin V - Cy3 (AnnCy3) conjugate emitting red fluorescence.

Solubility of *trans*-1

The solubility of ***trans*-1** in water was determined spectrophotometrically based on the absorbance at the absorption maximum of the ***trans*-1** saturated solution in water diluted 30 times and the absorbance of the standard 0.015 mg/mL solution of ***trans*-1** in water, which were equal to 0.93 and 0.72, respectively (**Figure S20**).

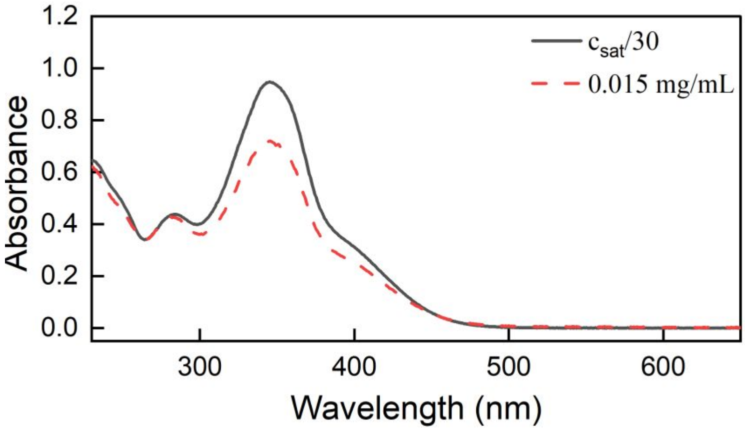

Figure S20. UV-Vis spectra of *trans*-1 saturated solution in water diluted 30 times (solid black line) and the standard 0.015 mg/mL solution of *trans*-1 in water (dashed red line).

ST1. Evaluation of exchange-correlation functional.

The validation process was carried out on the ***trans*-1** system, where the starting point of functional optimization was B3LYP also applied in the geometry optimization procedure. To ensure agreement with the empirical data, the excitation energies were derived with the use of an implicit COSMO solvation model, with DMSO as a chosen solvent. The SCF convergence parameters were set as in the geometry optimization procedure, similar to the used basis set.

Table S5. The excitation energies, oscillatory strengths and corresponding wavelengths obtained from TD-DFT calculations with B3LYP as an exchange-correlation functional.

TD-DFT (B3LYP) calculations			
Excitation	Ω [eV]	*f*	λ [nm]
1	2.903	0.0128	427.1
2	3.100	1.1334	400.1
Experimental	–	–	348.4

For the B3LYP, like for the tested functionals, the first two excitation energies (Ω_I) were calculated. The results of that initial procedure, including excitation energies and oscillator strengths f_I, are presented in **Table S5**. Vertical excitation of the electron to π^* orbital leads to a change in the electron distribution responsible for further geometrical distortion of the molecule to the *cis* configuration. The chosen transition is allowed by the selection rules thus it is characterized by high oscillator strength.

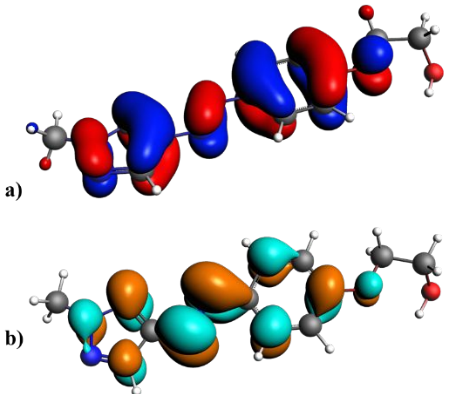

Figure S21. Visualization of the molecular orbitals involved in the transition predicted by TD-DFT(B3LYP) calculations a) the orbital 65 (HOMO) b) the orbital 66 (LUMO).

Error! Reference source not found. shows that the desired electronic transition most probably corresponds to a second excitation from TD-DFT(B3LYP) calculations, due to its highest oscillatory strength. Additionally, the visualizations of the involved in the transition orbitals (65 and 66; HOMO and LUMO, correspondingly), which are presented in **Figure S21**, indicate that the excitation has a strong $\pi \rightarrow \pi^*$ character. Despite predicted character and probability are correct, the wavelength λ of the

chosen excitation obtained from used procedure, significantly differs from the experimental value which equals to 348.4 nm. This result justified the necessity to perform the validation procedure.

In the validation procedure, a variety of different kinds of exchange-correlation functionals were applied in TD-DFT calculations of **1** transitions. The whole list of υ_{xc} used together with the results obtained using each methodology are presented in **Table S6Error! Reference source not found.**.

Table S6. The results obtained from the exchange-correlation functional validation of the TD-DFT vertical transitions calculations performed on 1.

TD-DFT calculations results				
$\boldsymbol{\upsilon_{xc}}$	**Orbital no.**	$\boldsymbol{\Omega}$ **[eV]**	$\boldsymbol{f}$	$\boldsymbol{\lambda}$ **[nm]**
B3LYP	2	3.100	1.1334	400.1
		**LDA**		
Xonly	2	2.752	1.1010	450.6
		**GGA**		
BP86	2	2.822	1.0917	439.4
BLYP	2	2.800	1.0697	442.9
PBE	2	2.822	1.0956	439.3
		**Hybrid**		
PBE0	2	3.197	1.1698	387.9
B3LYP*	2	3.026	1.1243	409.8
B1LYP	2	3.167	1.1395	391.5
KMLYP	2	3.521	1.1680	**352.2**
O3LYP	2	2.948	1.1385	420.6
BHandHLYP	2	3.472	1.1561	**357.1**
		**Hybrid Range Separated**		
CAM-B3LYP	2	3.395	1.1482	365.2
		**Hartree-Fock**		
HF	2	3.845	1.0621	322.5
Experimental	–	–	–	**348.4**

Error! Reference source not found. Table S7 presents the results obtained from the calculations with different hybrid functional excitation energies, corresponding wavelengths, and the percentage of used Hartree-Fock (HF) exchange energy. With an increasing contribution of considered HF part in the functional formulation the obtained values agree more and more with the experimental value. Therefore, considering the exact HF exchange energy describing the energy of the system coming from the Pauli principle is assumed as crucial in the description of the vertical transition where one electron is transferred to the unoccupied orbital, creating the situation where two electrons in the system have the same spin. The results closest to the experimental value were given by TD-DFT with KMLYP and BHandHLYP functionals, which had the highest HF energy percentage, 55.7%, and 50.0% respectively.

Table S7. The comparison of the excitation energies obtained from the calculations with different hybrid functionals.

υ_{xc}	**HF exchange energy contribution [%]**	Ω **[eV]**	λ **[nm]**
O3LYP	12.0	2.948	420.6
B3LYP*	15.0	3.026	409.8
B3LYP	20.0	3.100	400.1
B1LYP	25.0	3.167	391.5
PBE0	25.0	3.197	387.9
BHandHLYP	50.0	3.472	**357.1**
KMLYP	55.7	3.521	**352.2**
Experimental	–	–	**348.4**

As can be seen in **Error! Reference source not found. Table S7** the application of more complex functionals like CAM-B3LYP (a range-separated functional with corrected asymptotic), does not improve the TD-DFT calculations results, thus the validation procedure ended on the group of hybrid functionals. Because of less general applicability of the KMLYP which is rather unique exchange-correlation functional, the BHandHLYP was finally chosen to analyze the electronic excitation of the considered cisplatin derivatives.

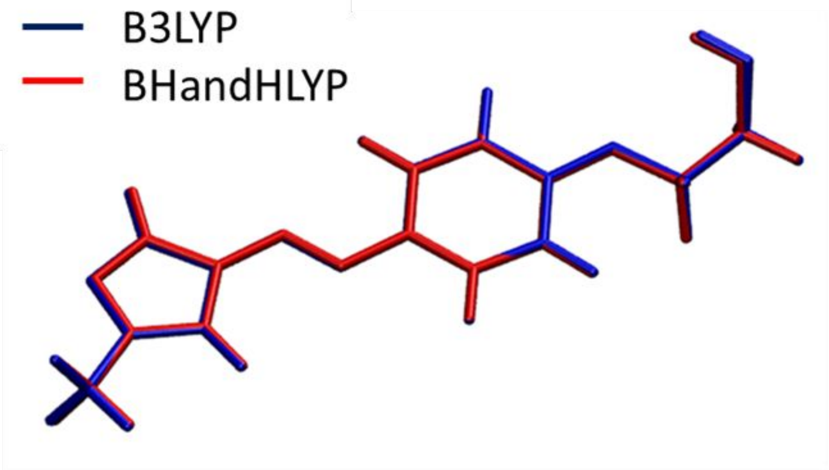

Figure S22. Alignment of ground state geometries obtained from the DFT calculations performed with B3LYP and BHandHLYP exchange-correlation functionals.

In the computational scheme used, all TD-DFT(BHandHLYP) calculations were performed on the structures obtained from the geometry optimization procedure, where the B3LYP was used as an exchange-correlation functional. It is reasonable to assume that if the ground state geometries of both B3LYP and BHandHLYP DFT are similar, the mixed computational procedure used will not affect obtained transitions, because the SCF procedure will be performed on the orbitals localized approximately on the ground state atomic positions. To quantitatively and qualitatively examine the difference between the geometries from B3LYP and BHandHLYP geometry optimizations, the proper calculations were conducted with the use of the same procedure as described above. The Root-Mean-

Square Deviation (RMSD) parameter is 0.054. **Figure S22** shows the alignment of both obtained structures.

ST2. Comparison of 1 and azobenzene

For both considered photoswitchable systems change of the *trans-cis* configuration leads to a decrease of the *end-to-end* length, defined as the distance between the carbon atoms at the para positions of aromatic rings in azobenzene, and between the carbon atom at the para position of the phenyl group and the nitrogen atom at 2nd position of pyrazole in **1**. There is a notable difference between the space occupied by each isomer, which may be important for instance where molecule's volume and length is the main structural feature responsible for its biological activity. The difference in the *end-to-end* distance between the photoisomers of **1** is significant and equals to □2,0Å (in azobenzene □2,7Å) indicating the possible use of the proposed AAP derivative in photopharmacological applications.

Table S8. The comparison of a selected structural properties, obtained from DFT/B3LYP geometry optimization calculations for trans-cis isomers of azobenzene and *cis/trans* photoisomers of 1.

	**azobenzene**		**1**	
	trans	***cis***	***trans***	***cis***
End-to-end distance [Å]	9.081	6.338	8.529	6.519
θ_{CNNC} [°]	179.400	8.562	179.18	0.328
$\lvert\mu_D\rvert$ [D]	0.056	4.884	5.029	8.006
$\Delta E_{\mathrm{rel}} \left[\frac{\mathrm{kcal}}{\mathrm{mol}}\right]$	0	9.91	0	9.83
$E_{\mathrm{solv}} \left[\frac{\mathrm{kcal}}{\mathrm{mol}}\right]$	− 3.60	− 7.43	− 16.06	− 20.42

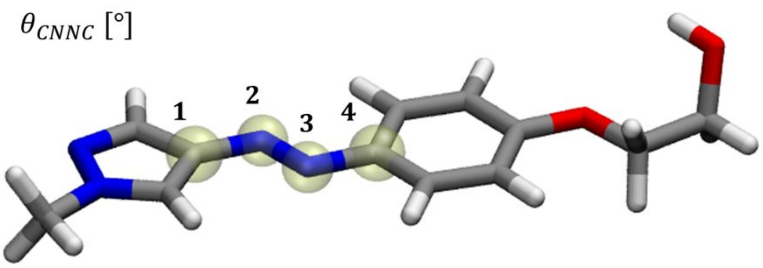

Figure S23. Visualization of the θ_{CNNC} dihedral angle in the 1 structure.

Another structural feature investigated by the geometry optimization was the dihedral angle, defined for the **1** molecule as an angle between the carbon atom in the 4th position of pyrazole, two nitrogen atoms

of the diazo bond, and attached carbon atom from the benzene ring (**Error! Reference source not found.Figure S23**). The data shown in **Table S8Error! Reference source not found.** indicate, that the *trans* and *cis* isomers both have almost exemplary values of θ_{CNNC} □180° and □0° respectively. It is worth noting that the difference between the θ_{CNNC} for ***cis*-1** and *cis*-azobenzene, can result from lower steric limitations in ***cis*-1**.

One of the main disadvantages of azobenzene limiting its use not only in photopharmacology but also in other fields of chemistry, is the thermal instability of its *cis* isomer, which thermally converts to a more favorable *trans* configuration. The relative values of the azobenzene and **1** systems energy, E_{rel}, (**Table S8Error! Reference source not found.**) provide information about the difference in the stability of the respective ***trans*-1** and ***cis*-1** isomers. In both systems, the *cis* isomer has higher energy than the *trans* one. It results from the steric repulsions, increasing with lowering the distance between the aromatic rings in both azobenzene and **1**. Change in the energy due to photoisomerization is almost identical for the azobenzene and **1**. The energy changes are different for the considered models which can be an effect of distinct optimal geometries (in the case of *cis* isomer), higher stabilizing delocalization effect within the molecular structure. Not negligible can be also a contribution of the solvation effect, which was quantitatively presented in **Table S8** as E_{solv}. As can be observed the proposed **1** structure is more soluble than azobenzene according to more stabilizing solvation energy (more negative values). Interestingly, ***cis*-1**, like *cis*-azobenzene, shows greater stability through solvation than and the *trans* isomer. Such a difference is probably related to the higher exposition of the nucleophilic azo-bonding nitrogen atoms to the water environment.

The difference in E_{solv} stabilizing the two isomers points to another important structural property that can lead to a potential difference in their bioactivity and depends on the azo bond configuration. The system rearrangement imposed by photoisomerization results in different charge distribution within the structure which leads to different chemical activity of the compound and for instance different solubility, observed for considered systems. The values of dipole moment, μ_D, the parameter related to mentioned charge distribution, calculated for both isomers of azobenzene and **1** (**Table S8**) **Error! Reference source not found.**indicate that the photoisomerization from *trans* to *cis* configuration leads to an increase in compound polarity (higher $|\mu_D|$). According to the obtained data, a change of the dipole moment module is lower for **1** ($\Delta|\mu_D| = 2.977$ D) than for azobenzene ($\Delta|\mu_D| = 4.828$ D), which can be related to the fact that the charge distribution within the **1** molecule is more heterogenous, due to the presence of heteroaromatic pyrazole ring and the ether substituent, thus the strong exposition of nitrogen lone pairs in ***cis*-1**, responsible for higher $\Delta|\mu_D|$ associated with photoisomerization, is partially neutralized by the effects from other atoms. For instance, the lone pair of one of the pyrazole's nitrogen atoms is directed almost oppositely to the azo bond moiety, likewise the that of the hydroxyl group at the end of the molecule.

ST3. Conformational space screening - Molecular Dynamics studies as the evaluation of the dynamic stability of $Pt(1)_2$ group

In the performed MD procedure, the analyzed structures were put in the cubic computational box of dimensions 25 × 25 × 25 Å with imposed periodic boundary conditions. The simulation was carried out in canonical ensemble (NVT), which allows for an accurate description of conformational space. The time step of the simulation was 1 fs, and the temperature was set to 300 K. The full length of the simulation for every considered compound lasted 25000 fs. All MD simulations were conducted in the gas phase.

The plot, presented in **Figure S24Error! Reference source not found.**, shows the energy changes during the MD simulation. Interestingly, at all time points the energy of ***cis*-2** is higher than that for the ***trans*-2** isomer.

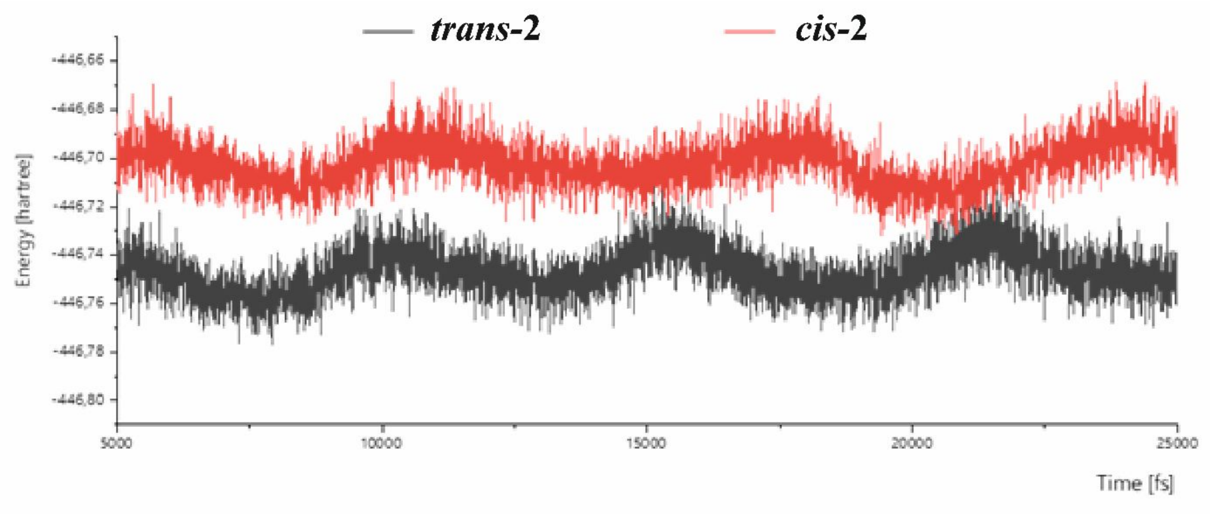

Figure S24. The comparison of the internal energy changes during the 20000 steps of the performed MD simulation.

Further analysis of the obtained trajectories indicates that both isomers of the ligands in the complex preserve relatively stable conformations during the whole simulation. The internal energy of the system oscillates with similar frequency in the range of 0.07 hartree (43.93 $\frac{\text{kcal}}{\text{mol}}$) and 0.06 hartree (37.65 $\frac{\text{kcal}}{\text{mol}}$) for *trans* and *cis* isomers, respectively. The obtained energy oscillations corresponding to the conformational changes indicate that there is no particular preferable geometry in the considered systems.

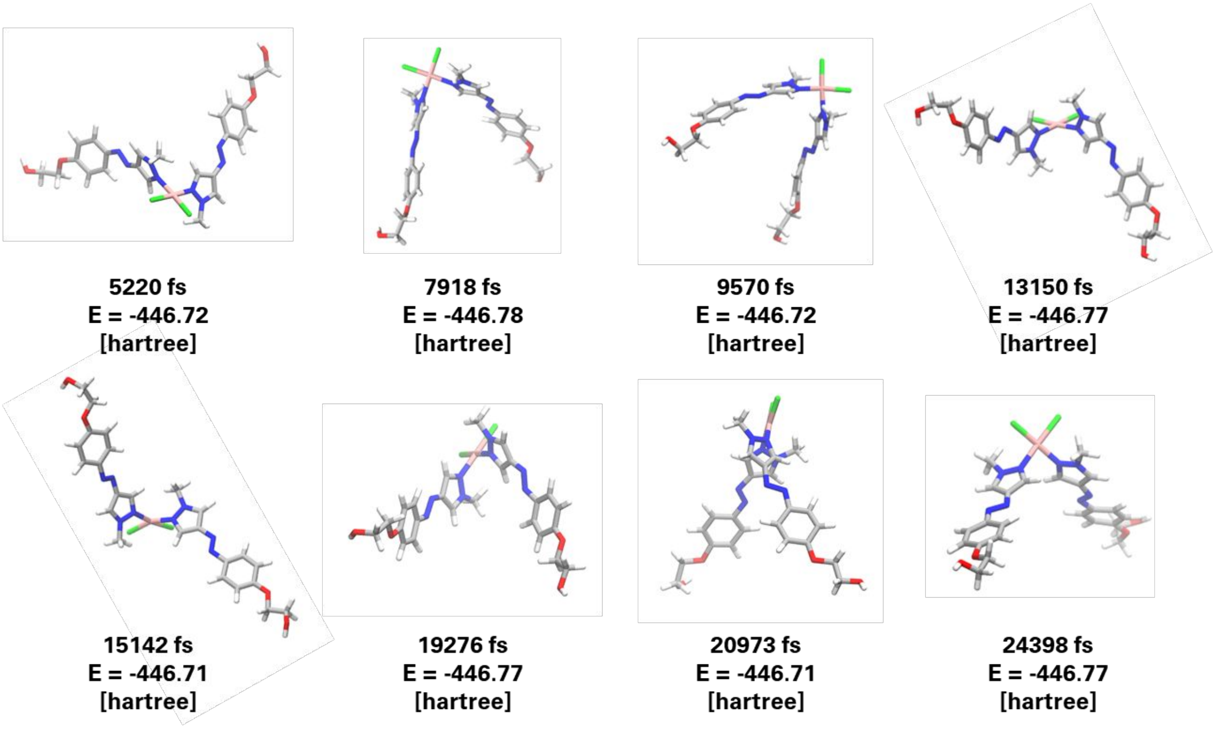

Figure S25. Conformations with the highest and lowest energies obtained from the *trans*-2 conformational space screening.

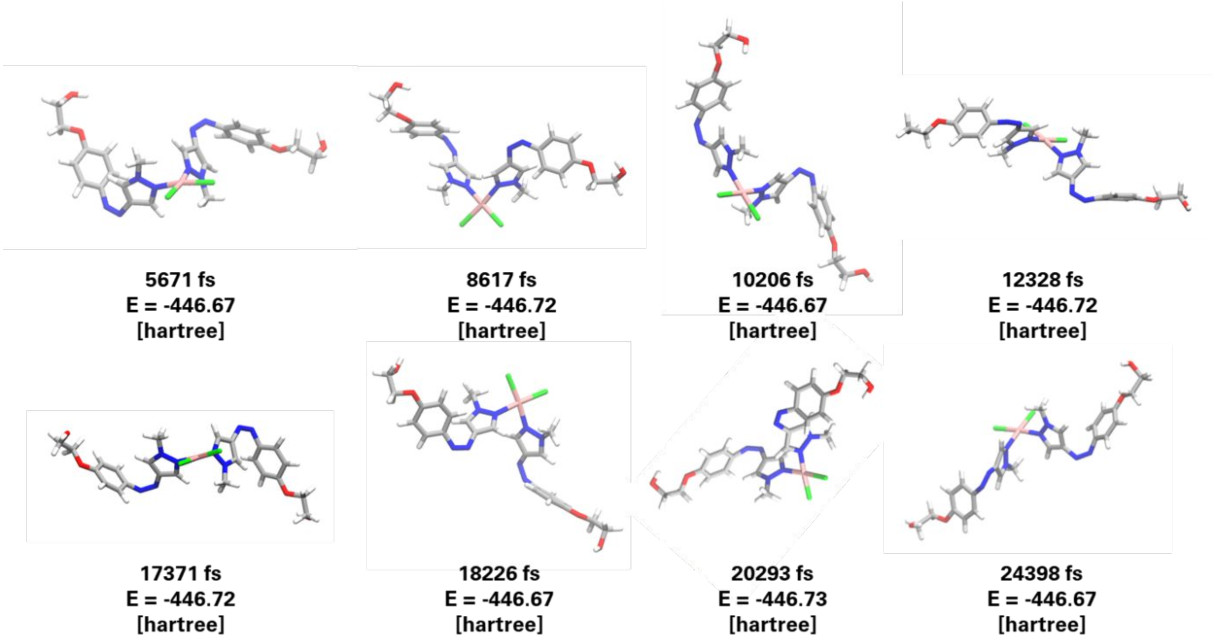

Figure S26. Conformations with the highest and lowest energies obtained from the *cis*-2 conformational space screening.

To present mentioned similarities between the considered conformations indicated by the conformational space screening, the geometries with the highest and lowest energies were compared (**Figure S25Error! Reference source not found.** and **Figure SError! Reference source not found.25**). It should be mentioned that during the MD simulation ***trans*-2** never adopts the geometry from the optimization process in contrast to the *cis* isomer, for which the geometries from the trajectory

are analogical to the initial structure (**Error! Reference source not found.**, please compare the supplemented movies).

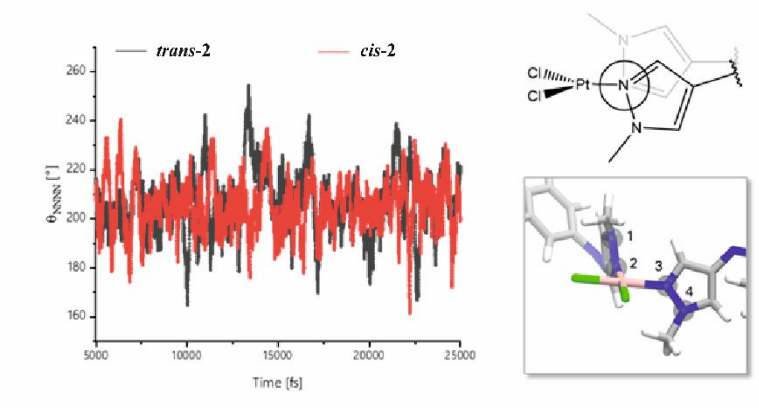

Figure S27. The plot presenting the analyzed hypothetical dihedral angle θ_{NNNN} between the four nitrogen atoms of the pyrazole heteroaromatic rings for both *trans*- and *cis* complex photoisomers.

Substitution of the 1st position of pyrazole limits the rotations of the heteroaromatic rings along the Pt-N bond resulting in their almost mutual parallel orientation in both photoisomers as confirmed by the MD simulation. Any change in that orientation leads to the energetical cost, destabilizing the system via the steric hindrance. The analysis of the plot of θ_{NNNN} dihedral angle between four nitrogen atoms of the pyrazole rings as the function of the timestep is presented in **Figure S27Error! Reference source not found.**. Its value ranges from approximately 90 to 240° as the methyl groups inhibit further rotation. The average values of θ_{NNNN} are equal to 206.60 and 204.82° for *trans* and *cis* photoisomers, respectively.

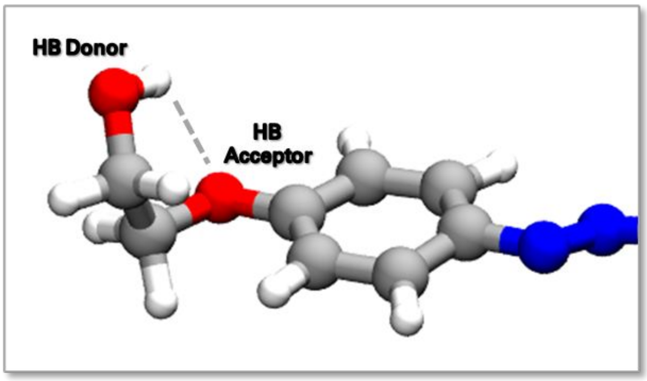

Figure S28. Considered hydrogen bonding between the hydroxyl group at the end of the 1 moiety and the ether oxygen in the Pt(1)$_2$ complex.

Another interaction possible within the complex is the hydrogen bonding (HB) between the hydroxyl group and the ether oxygen as a hydrogen acceptor. The considered HB was schematically shown in **Figure SError! Reference source not found.28**. The MD trajectories for *trans* and *cis* isomers just

indicate the possibility of such an interaction, however, they do not prove its existence or its quantitative characteristic. The possible HB interaction could not be directly related to any energy trend observed from the MD trajectories for both isomers.

The stability of the planar geometry of the **1** ligands during the simulation was evaluated. Firstly, the dynamic stability of the *trans/cis* 1 moieties was analyzed by plotting the corresponding dihedral angle (**Figure S29Error! Reference source not found.)**.

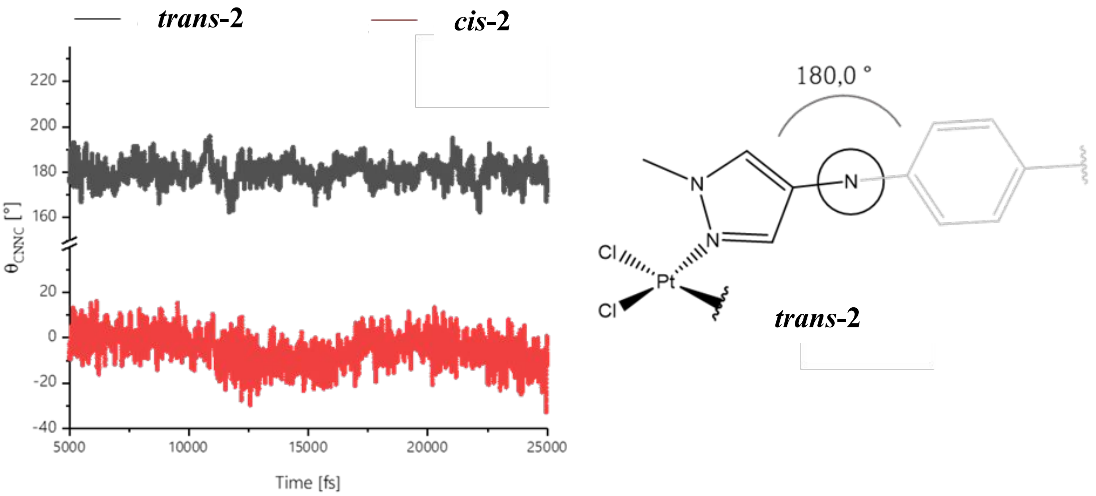

Figure S29. Comparison of the average values of the dihedral angle corresponding to the diazo bond configuration for both isomers of the Pt(1)$_2$ systems.

The configurations of the diazo bonds within the **1** moieties are preserved during the whole MD simulation and the *trans* and *cis* configurations of both **1** moieties within one complex do not differ significantly which could be expected considering the high symmetry of the compounds and lack of any non-heterogenous interactions between the *N*-donating ligands. More interestingly, the lower oscillation of the θ_{CNNC} values of the *trans* photoisomer results in its higher stability compared to the more sterically hindered *cis* photoisomer. Besides described difference, both photoswitchable ligands are considered as stable in the simulated temperature.

The dynamical stability of the planar geometry within the coordination center of the complex was analyzed. To investigate how mutual position of the atoms attached to the platinum atom change in time at 300 K, the changes of the θ_{ClPtNN} dihedral angle between the plane defined by chlorine, platinum and nitrogen atoms (atoms 1,2 and 3 in **Figure S30Error! Reference source not found.**) and the plane defined by two coordinated nitrogen atoms and a the platinum atom were calculated (atoms 2, 3, and 4 in **Figure S30Error! Reference source not found.**). This angle is expected to have the value around the 180° considering the planar geometry of the coordination center.

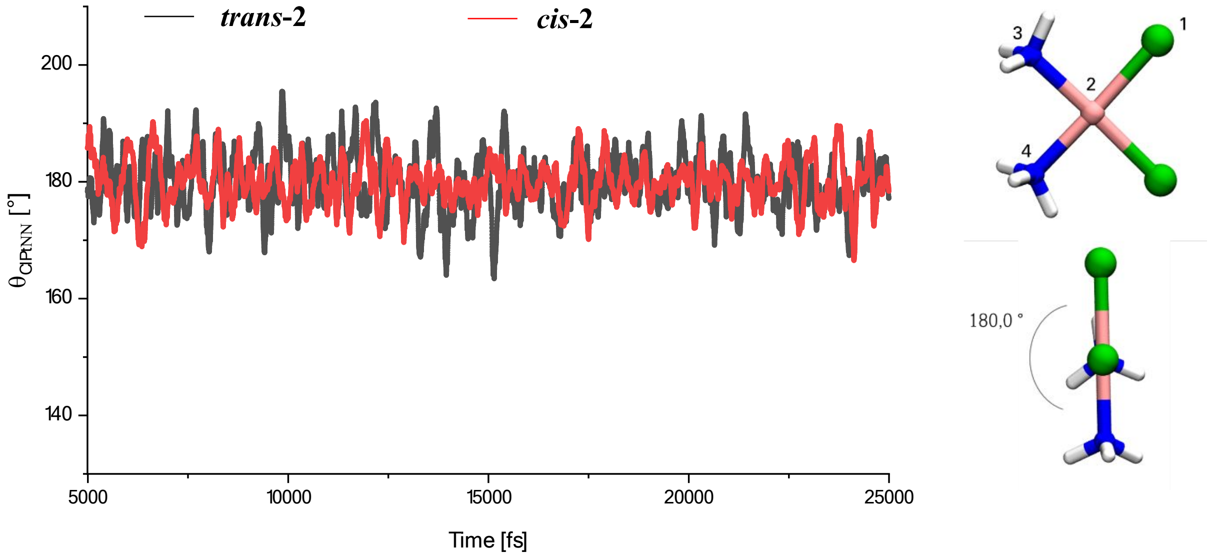

Figure S30. The dependence of the values of the θ_{ClPtNN} dihedral angle on time indicating stability of the coordination center planarity in the Pt(1)$_2$ system of both photoisomers. The atoms forming θ_{ClPtNN} are shown on the right panel. Cisplatin structure was used for simplification.

The analysis of θ_{ClPtNN} fluctuations indicates the stability of the planar geometry during the whole MD simulation. The average values for each angle vary ± 15° around the value of 180°. The slightly higher oscillations of the dihedrals were observed within the ***trans*-2** molecule. The average values of all analyzed dihedral angles are presented in **Table S9**. The upper index corresponds to the number of the ligand, while the subscript defines the atoms for which the dihedral angle was defined.

Table S9. The comparison of the obtained values of average dihedral angles describing the dynamic properties of the Pt(1)$_2$ systems in both photoisomers.

	**Pt(1)$_2$**	
	trans	***cis***
θ^1_{CNNC} [°]	179.94	− 3.37
θ^2_{CNNC} [°]	180.07	− 6.82
θ_{NNNN} [°]	206.60	204.82
θ^1_{ClPtNN} [°]	180.00	179.74
θ^2_{ClPtNN} [°]	180.00	179.95

References

(1) Wolff, J. E. A.; Trilling, T.; Mölenkamp, G.; Egeler, R. M.; Jürgens, H. Chemosensitivity of Glioma Cells in Vitro: A Meta Analysis. *J. Cancer Res. Clin. Oncol.* **1999**, *125* (8–9), 481–486. https://doi.org/10.1007/s004320050305.
